# Revolutionizing Brain Research Using Portable MRI in Field Settings: Public Perspectives on the Ethical and Legal Challenges

**DOI:** 10.1007/s12152-025-09606-4

**Published:** 2025-07-26

**Authors:** Molly K. Madzelan, Frances Lawrenz, Susan M. Wolf, Francis X. Shen

**Affiliations:** 1 Federation of Associations in Behavioral & Brain Sciences, Washington, DC USA; 2 University of Minnesota, Minneapolis, MN USA

**Keywords:** Neuroethics, Research ethics, Portable magnetic resonance imaging (MRI), Neuroimaging, Therapeutic misconception, Community engagement

## Abstract

**Introduction:**

New, highly portable MRI (pMRI) technology promises to revolutionize brain research by facilitating field-based studies that can expand research to new settings beyond the traditional MRI suite in a medical center. At this early stage of development, understanding public knowledge and attitudes about pMRI research is crucial.

**Objective:**

In this article we present the first empirical study of the general public’s willingness to participate in pMRI research, and their perceptions of expected benefits and concerns.

**Methods & Results:**

We conducted a nationally representative online survey (N = 2,001) administered Aug. 15-31, 2022. We found that respondents were overwhelmingly willing to participate in pMRI research, with no significant differences between five key demographic sub-groups: rural residents, older adults (65+), Hispanics, non-Hispanic Blacks, and those economically disadvantaged. Respondents saw many potential benefits (e.g., follow-up information about the study’s results) and few concerns (e.g., insufficient payment) associated with participating.

**Conclusion:**

Such high public interest in participating confirms the importance of developing ethical guidance for pMRI research now, before that research rapidly expands. The results speak to the importance of minimizing the therapeutic misconception in pMRI research, as the survey reveals gaps in participant knowledge about the capabilities and limitations of pMRI devices to provide clinically informative scans. Our data showed that a lack of trust in scientists can reduce likelihood of participation, and thus researchers will need to engage participant communities to fully realize the potential of pMRI research to reach remote and historically underrepresented populations.

**Supplementary Information:**

The online version contains supplementary material available at 10.1007/s12152-025-09606-4.

## Introduction

Since its invention in the late 1970s, magnetic resonance imaging (MRI) has transformed neuroscience research and raised ethical, legal, and societal issues (ELSI). But to date MRI research has typically required research participants to travel to a large MRI scanner, usually located in a hospital or university research facility [1, 2]. Requiring participants to travel to the scanner has contributed to the well-recognized problem of insufficient geographic, racial, cultural, and socioeconomic diversity in MRI research and databases [3].

The advent of highly portable MRI (pMRI) technologies will disrupt the traditional paradigm of MRI research, raising novel ELSI issues [3]. MRI research will soon be conducted both in the lab *and* in the field, as smaller, cheaper, and much more portable devices are used to scan participants in locations such as a school gymnasium, a mobile MRI van that makes house calls, a community center, and the sidelines of a sporting event [4]. pMRI is already being used to scan participants in a mobile van outside their homes [5] and in an ambulance [6, 7]. In addition to new locations, the user-friendly interfaces of portable MRI technologies will allow for new types of investigators to conduct research, including citizen scientists and community groups [3].

pMRI research in new locations, with new participants, and by new investigators will raise significant ELSI challenges [3]. Addressing these challenges now is critical, before pMRI research proliferates. An essential step in anticipatory governance of emerging technology is to understand the public’s attitudes [8, 9]. This paper presents the first study of public opinion on pMRI research, filling an important empirical gap and informing development of ethical and legal guidelines for rapidly expanding pMRI research.

Our approach follows the tradition of pragmatic neuroethics and bioethics analysis. As evidenced in the title of a 2004 article by Miller and Fins, “[p]rotecting human subjects in brain research” has been a core concern in pragmatic neuroethics scholarship for two decades [10]. Because “pragmatic ethical analysis is informed by relevant empirical inquiry,” pragmatic neuroethics has integrated empirical research such as utilizing lived experience [11], focus groups, and surveys to inform the development of ethical safeguards for neuroscience research [11, 12]. From a pragmatist neuroethics perspective, one of the values of empirical research is that it “makes us aware of overlooked or underappreciated issues, thus providing additional lenses through which to view familiar problems” [13]. This study adopts such a lens, utilizing a survey of the general public to help identify potential ethical concerns and societal benefits associated with pMRI research.

We conducted a nationally representative online survey of 2,001 respondents from Aug. 15–31, 2022, powered to generalize to the U.S. public. Because the features of pMRI give it the potential to reach rural populations and improve neuroscience research with underrepresented populations, we also powered the survey to facilitate sub-group comparisons across five relevant demographic sub-groups: self-identified rural residents, older adults (65+), those who self-identified as non-Hispanic Blacks or Hispanics/Latinos, and those living below the federal poverty line (calculation described below). This survey was part of a larger project on “Highly Portable and Cloud-Enabled Neuroimaging Research: Confronting Ethics Challenges in Field Research with New Populations,” funded by the U.S. National Institutes of Health (NIH) Brain Research through Advancing Innovative Neurotechnologies (BRAIN) Initiative (RF1MH123698). Preliminary results from the survey data reported here were utilized by the project’s Working Group to inform consensus recommendations on addressing ELSI issues in pMRI research [14].

We designed the survey to focus on three key sets of questions that should shape ethical and legal guidance for emerging pMRI research. First, we queried public willingness to participate in research. If the public is not willing to participate in research with a new technology, significant investment in ethics guidance for that research may be misplaced. On the other hand, if the public is eager to participate, formulation of ethical guidance becomes urgent. We thus started the survey by asking about respondents’ willingness to participate in pMRI research, and evaluating whether there were differences by key demographics. Second, after establishing baseline willingness to participate, we wanted to explore the factors that shape respondents’ attitudes toward pMRI research. Thus, we next examined what potential benefits and harms respondents anticipated from pMRI research, again analyzing whether these assessments of benefits and harms varied by key demographics. Third, given the importance of expanding and diversifying participation in human neuroimaging research [15], we sought to understand what factors, including trust in researchers, appeared to increase or decrease likelihood of participation.

## Research Design and Methods

### Compliance with Ethical Standards

This research was approved by the Institutional Review Board of the University of Minnesota (Study ID: 00015482). Prior to starting the survey, all respondents provided informed consent.

### Sampling Strategy to Ensure Representativeness

Surveys are regularly used in empirical bioethics and neuroethics scholarship to investigate expert, public, patient, and medical provider perspectives on emerging technologies and the ethical issues they may raise [16, 17]. Survey methods have been utilized to examine expert views on neurotechnology [18–20], MRI research participants’ expectations regarding the likelihood of abnormal scan results [21], and patient and provider attitudes on the clinical use of functional MRI in treating major depression [22].

For this public survey, we sought a representative sample in order to make generalizable claims about national public attitudes [23]. Historically, the standard means of obtaining a representative sample was probability sampling through random digit dialing [24]. However, survey research has transformed significantly [25], and probability sampling has been challenged by high nonresponse rates and incomplete coverage of the population, leading to inaccurate results [25, 26]. As one expert observed, “survey research is in a state of crisis” [27].

Given high nonresponse rates to traditional probabilistic sampling methods using telephone calls, researchers have increasingly turned to non-probability sampling designs [28]. Non-probability methods do not rely on random sampling from the population of interest and instead use alternative methods to develop a sample that will support generalized inferences about the population of interest. Non-probability sampling recruits individuals to fill each needed demographic group in order to match the sample to the population of interest. For example, if the population of interest includes 15% of Group A, then the survey researchers will recruit 15% of participants from Group A. A significant limitation of this approach, however, is that it is not clear if those members of Group A who have opted-in to the survey are representative of all members in Group A.

The empirical research comparing results of non-probabilistic versus probabilistic sampling is both encouraging and concerning. On one hand, in 2013 the American Association for Public Opinion Research Task Force on Non-Probability Sampling concluded that non-probability sampling techniques can be effective under certain circumstances and if careful attention is paid to sampling methodology [29]. For instance, a study of 17 online panels found that they produced consistent estimates of outcomes such as life satisfaction and purchasing behavior [30]. Of relevance for our work, a line of scholarship has found that non-probabilistic sampling methods may be beneficial for including hard-to-reach populations [31, 32]. On the other hand, research has also found that some non-probability samples perform worse than comparable probability-based samples [33].Potential sources of bias that emerge when using non-probability online sampling include potential differences between those with digital access (required for participation in our survey) and non-digital populations [34].

Although these limitations need to be acknowledged (as we do below), carefully designed non-probability sampling is an improvement upon common survey methods approaches in neuroethics empirical survey research. Much neuroethics scholarship has not used probabilistic methods. For example, Amazon Mechanical Turk (MTurk) has been utilized by neuroethics researchers to recruit respondents for studies of public attitudes toward neurotechnology [18] and enhancement [35, 36]. But previous research has found that participants recruited via MTurk are not representative of the U.S. public [37, 38]. MTurk samples are typically younger, more educated, more liberal, and less racially diverse than the U.S. general population [36, 39–41].

To be sure, the neuroethics literature is starting to address this methodological concern. For example, a recent neuroethics study examining “perceptions of implanted neural devices among groups underserved by prevailing neuropsychiatric treatment approaches” [42] developed a nationally representative sample by utilizing a probability-based panel, and this study over-sampled on target demographic sub-groups relevant to the study: Black, Hispanic, and rural Americans. Our study similarly adopts this approach of over-sampling of key sub-groups.

### Survey Development Through Pilot Surveys and Working Group Feedback

Asking respondents how likely they are to participate in research with pMRI requires supplying context, as the general public is otherwise unlikely to understand what pMRI is and what participation in such research involves. To address such challenges, empirical studies in bioethics have utilized vignette-based questions [43]. The use of hypothetical vignettes has strong grounding in the social sciences [44, 45]: “Unlike attitudinal scales that ask direct questions about values and beliefs, vignettes offer an approach that assesses individuals’ attitudes or values *in a contextualized scenario or situation*” [43]. The vignette approach has been used in studies of physicians’ use of MRI and clinical assessment in Neonatal Hypoxic-Ischemic Injury [46], and in assessing public attitudes towards cognitive enhancement [36].

We adopted the anchoring vignette approach and utilized an iterative Working Group (WG) process to develop a vignette that provided information in a way that survey participants could understand, while still communicating sufficient detail about the portable technology. The project’s interdisciplinary WG included 15 members, in addition to the Principal Investigators (PIs), “with expertise in neuroscience, neuroimaging, radiology, research ethics, community engagement, law, neurology, and artificial intelligence” [3]. At multiple meetings in the first two years of this project the WG advised on the design and refinement of the survey instrument.

At the start of the project, the WG participated in a modified Delphi process that allowed the PIs to generate an initial draft of the anchoring vignette and questions for the general public survey. We further refined the anchoring vignette through a series of 11 pilot studies conducted May – Dec. 2021 and through WG discussion of the pilot results over the course of three WG meetings. Pilot participants were recruited through Prolific, an online research platform that provides researchers with more representative samples than MTurk [47]. Each pilot survey presented different vignettes and questions, allowing us to test different approaches to assessing public willingness to participate in pMRI research and perceived benefits and concerns. For example, we used the pilot surveys to refine our attention check and knowledge check questions. We also pilot tested language choices to avoid biasing participant responses, and we reviewed multiple versions of the illustrative figure with the WG to ensure that what it depicted was consistent with the science of pMRI. We also tested versions of the survey with and without the figure, with no substantive difference in pilot results between the two conditions. The piloting process with WG feedback yielded final survey language that the project team and WG felt was clear, unbiased, and a realistic presentation of pMRI research. Regarding survey items that refer to values such as “trust,” we chose to let respondents define these terms for themselves in order to capture their subjective attitudes toward the pMRI scenario and ascertain what values they thought were at stake.

All survey questions were tested by the Flesch/Flesch–Kincaid Readability Test [48] online test tool [49] to help develop question text that met the guidelines for health information readability at a 9th grade level [50]. The pilot surveys were administered via Qualtrics.

### Final Survey Instrument

The full text of the final survey can be found in Online Resource 1. Only items relevant to the reported analyses are described in text. After reading an information sheet and providing informed consent, all respondents read the vignette and saw the figure reproduced in Box 1.
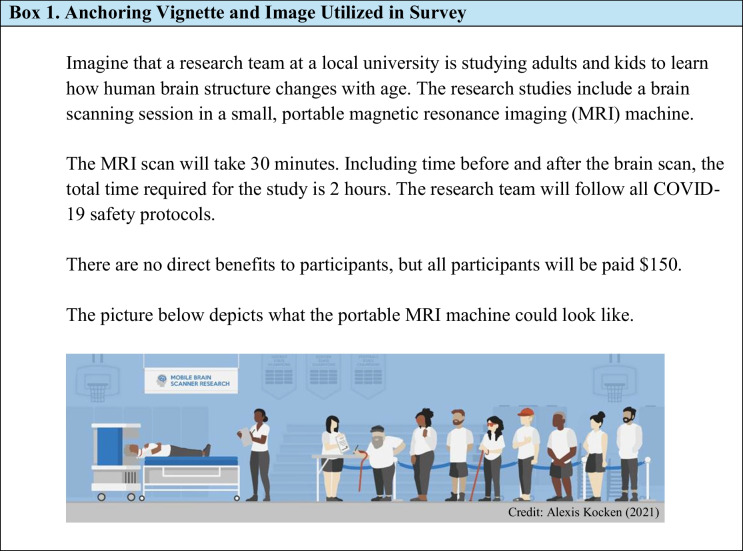


#### How Likely Is the General Public To Participate in Portable MRI Research?

Our first battery of questions asked how likely respondents would be to: (a) participate in pMRI research themselves, (b) encourage a close friend to participate, (c) allow an older or vulnerable adult to participate, and (d) allow a 7-year-old child to participate. The four questions were randomly presented to account for possible order effects. Responses were measured using a 5-point Likert scale: (1) certainly refuse, (2) probably refuse, (3) not sure, (4) probably participate, and (5) certainly participate.^1^

To further probe likelihood of participating in pMRI research, we presented respondents with nine factors that might increase or decrease their own likelihood of participating. Each factor was followed by the question, “… would this make you more or less likely to participate in this research study?” (see Table 1 for the full wording of each question stem). For these questions, respondents were only asked about their own likelihood of enrollment, not about a friend’s, vulnerable adult’s, or child’s likelihood of enrollment. Responses were measured using a similar 5-point Likert scale: (1) much less likely to enroll, (2) less likely to enroll, (3) neutral, (4) more likely to enroll, and (5) much more likely to enroll. Responses below the midpoint (3, neutral) indicated that the factor *decreased* the likelihood of enrollment in this kind of research, while responses above the midpoint indicated that the factor *increased* the likelihood of enrollment. These nine items were randomly presented to respondents to account for possible order effects.

**Table 1:** Full Wording of Survey Items: Influential Factors Battery

Would This Make You More or Less Likely to Participate in the Research Study?
If participating in the research study **included sending you a brain health report based on the MRI images at no cost**. [*report*]
If the research team **sent you a copy of your brain scan images after your participation**. [*scans*]
If the research team were able to **put the MRI machine in a van and come to your home to do the scan without requiring you to travel**. [*home*]
If the research team were able to **set up the portable MRI in a convenient location near your home** such as a community center. [*location*]
If the research was **led by a research team that consulted with members of your community before conducting the study**. [*community*]
If the research was **conducted by a research team that included racial or ethnic minority scientists**. [*minority scientists*]
If the research was **conducted by researchers from a private, for-profit company** not associated with a university. [*for-profit*]
If the research required **receiving an injection of a contrast material into your blood stream**. The contrast material does not color organs, and does not contain radiation. The contrast liquid inserted into the blood stream enables the technology to create clearer pictures of your brain. [*injection*]
If the research required you to **travel to and from a large hospital**. (You would be responsible for arranging your travel to and from the hospital.) [*hospital travel*]

*Notes:* In the survey itself, each statement was followed by the question, “…would this make you more or less likely to participate in the research study?” The label for each item in square brackets [] was not included in the survey question but is added here to correspond with the labels used in this article’s figures and regression tables

#### What Potential Benefits and Concerns Does the Public Perceive in pMRI Research?

In the next two sections of the survey, respondents were asked to consider potential benefits and concerns in pMRI research (Table 2). Respondents were presented with nine potential benefits and instructed to rate the importance of each one to them using the following 5-point Likert scale: (1) not important, (2) somewhat not important, (3) neither, (4) somewhat important, and (5) very important. Next, respondents were presented with 11 possible concerns and were again instructed to rate the importance of each one to them using the same 5-point Likert scale. The items were randomly ordered within each table.

**Table 2:** Full Wording of Survey Items: Potential Benefits and Concerns

Potential Benefits	Potential Concerns
After the research is completed, get follow-up information about the results of the study. [*follow-up info*]	Not enough payment for time and travel. [*payment*]
Learn if I have a medical condition that needs attention. [*learn condition*]	Brain scan might be used by insurance companies to raise my insurance rates. [*insurance*]
Learn more about your brain's health. [*learn brain*]	Researchers will not respect my rights or privacy. [*privacy*]
Financial payment for time and travel. [*payment*]	Brain scan will not be safe. [*safety*]
Desire to help others and benefit future patients. [*help others*]	Metal or electronic device in my body. [*metal in body*]
Contribute to scientific progress. [*science*]	Scared that I will find something wrong with my brain. [*something wrong*]
Access to medical treatment. [*treatment*]	Researchers cannot be trusted/not sure of their motivations. [*trust*]
Get to see a cool picture of my brain scan, even if not part of a brain health report. [*cool picture*]	Too busy and not enough time to participate in this research. [*time*]
Interesting thing to do. [*interesting*]	Brain scan would be too uncomfortable/I am claustrophobic. [*discomfort*]
	Research team will not understand or respect my cultural and community values. [*values*]
	Researchers may use the brain scan for mind control. [*mind control*]

*Notes:* Respondents were presented with two tables, one for benefits and one for concerns, which listed these items in a randomized order. The label for each item in square brackets [] was not included in the survey question but is added here to correspond with the labels used in this article’s figures and regression tables

#### Factors to Explain pMRI Attitudes

To explore possible factors driving the public’s attitudes about pMRI research, we asked respondents to self-report their income, age, race and ethnicity, and geographic location (large city, suburb, small city, rural area, or Tribal Lands) (see Supplemental Materials). In addition, we asked about respondents’ prior MRI experience to create a variable called *MRI experience* distinguishing between those who had an MRI scan before and those who had not. Our *MRI experience* question focused on traditional fixed MRI, as pMRI has only been utilized to date in a small number of research studies and clinical contexts.

We also utilized three items from the 12-item Mainous et al. validated trust in biomedical research (TBR) instrument [51]. The Mainous et al. TBR instrument is one of the most highly cited TBR scales [52]. To ensure that we deployed the TBR instrument consistently with previous research, we utilized questions verbatim. This included using the phrases “minority subjects” and “white subjects,” even though contemporary usage might be different. In our survey, respondents were asked to rate how strongly they agreed or disagreed with the following statements regarding medical research generally:

- To get people to take part in a study, medical researchers usually do not explain all of the dangers about participation. [*dangers*]
- Usually, researchers who make mistakes try to cover them up. [*cover-up*]
- Medical researchers act differently toward minority subjects than toward white subjects. [*perception of racial bias*]

Responses were measured using a 5-point Likert scale: (1) strongly disagree, (2) disagree, (3) neutral, (4) agree, and (5) strongly agree. The first two items – *dangers* and *cover-up* (*r* =.63) – were also combined to form a composite variable, *research skepticism*, assessing respondents’ skepticism about medical researchers’ intentions and honesty. The third item – *perception of racial bias* – measured respondents’ perceptions of racial bias among medical researchers.

We also asked respondents to self-report gender (male, female, other)^2^ and zip code. At the end of the survey, respondents were directed to the final page where they were thanked for their participation and given contact information for a member of the research team whom they could contact with any additional questions about this study.

### Recruiting Respondents for Nationally Representative Survey

#### Recruitment

We contracted with the survey opinion firm Verasight to recruit a nationally representative sample and administer the survey. Verasight used an iterative raking procedure to create a set of weights that were based on Current Population Survey benchmarks of age, sex, race and ethnicity, education, and region, as well as population benchmarks of 2020 presidential vote choice.^3^ Respondents were recruited and compensated directly by Verasight. “All respondents were recruited from the Verasight Community. The Verasight Community is composed of respondents who participated in prior Verasight surveys. These individuals were recruited through a combination of both random address-based sampling and online advertisements across search networks and social media platforms. To maintain the panel, Verasight uses a multi-step authentication to verify users. Panelists who exhibit low-quality response behaviors, such as straight-lining or speeding, are also removed and banned from participating in the panel” [53].

To determine an appropriate sample size for our planned analyses, we conducted an *a priori* power analysis following a standard formula in biostatistics [54]. To have sufficient power, we needed at least 1,067 respondents from the general public (we had 2,001) and to additionally ensure that we could compare across key demographic sub-groups we needed between 150–200 respondents in each, which we achieved. Sub-groups were defined as follows:

- **Rural**: Based on self-reported location, we labeled as Rural those respondents who selected “Rural area” in response to the question “Which of the following best describes the place where you now live?”
- **Older Adults**: Based on self-reported age data, we defined “older adults” as respondents who were 65 years of age or older on the day of survey participation. Age 65 is a cut-off that NIH institutes/centers and researchers frequently use to study aging populations, for instance in the National Health and Aging Trends Study [55].
- **Hispanic**: Based on self-reported ethnicity, we labeled as Hispanic those who self-identified as Hispanic of any race.
- **Non-Hispanic Black (“Black”)**: Based on self-reported ethnicity and race, we coded as Non-Hispanic Black those who indicated they were not Hispanic and then checked only the “Black or African American” option when asked to describe their race. Those who selected more than one race were coded as Multi-racial.
- **Economically Disadvantaged**: The NIH category of “individuals from disadvantaged backgrounds” includes “individuals who come from a family with an annual income below established low-income thresholds” as set by the U.S. Federal Poverty Guidelines Used to Determine Financial Eligibility for Certain Federal Programs [56]. Respondents were labelled as Economically Disadvantaged based on their reported income, zip code/state, and household size.^4^

#### Final Sample and Demographics

Following the pilot studies described above, data collection for the main study began on Aug. 15, 2022, and concluded on Aug. 31, 2022. The survey was administered by Verasight using Qualtrics. In total, 2,001 individuals in the U.S. took part in the final study, with oversamples on the five key demographic sub-groups. Weighted demographic information on the final sample is reported in Table 3, which also compares demographic percentages in our sample to U.S. 2020 Census percentages. 51% of the sample identified as female, 48% identified as male, and approximately 1% selected “other.” An exact comparison to Census data is not possible as the U.S. Census asks about biological sex, not gender. However, our sample numbers closely match those reported in the 2020 Census: 52% female and 48% male. In terms of self-identified ethnicity, 17% of our sample identified as Hispanic while 83% did not, which aligns with the 2020 Census reporting 19% of the general U.S. population as Hispanic and 81% as non-Hispanic. The sample was also representative with respect to self-identified race (65% White in our sample, compared to 62% nationally; 13% Black or African American in our sample, compared to 12% nationally). Our sample did deviate from the 2020 Census in terms of Medicare status, with 43% of our sample reporting that they were covered by Medicare compared to 24% of the 2020 U.S. general population. However, our sample was similar to the general population in terms of overall insurance status, 89% insured in our sample versus 91% insured nationally.

**Table 3:** Demographic Information for the Final Sample, with Corresponding 2020 U.S. Census Information

Demographic Variable	Sample Percentage	Census Percentage
Gender^a^		
Female	51%	52%
Male	48%	48%
Other	1%	n/a
Age		
18–20 years	5%	5%
21–44 years	43%	41%
45–64 years	29%	32%
65 years and older	22%	22%
Race		
White	65%	62%
Black	12%	12%
Asian	6%	6%
Native American	0%	1%
Pacific Islander	0%	0%
Other	10%	8%
More than one	6%	10%
Ethnicity		
Hispanic	17%	19%
Not-Hispanic	83%	81%
Geographic Area		
Not rural	83%	88%
Rural	17%	12%
Education		
Some high school or less	4%	10%
High school graduate or GED	34%	28%
Some college, no degree	17%	17%
2-year or associate degree	10%	10%
4-year or bachelor degree	20%	22%
Post-graduate degree	15%	13%
Income		
Less than $15,000	10%	10%
$15,000 to $24,999	11%	8%
$25,000 to $34,999	8%	8%
$35,000 to $49,999	8%	11%
$50,000 to $74,999	23%	17%
$75,000 to $99,999	15%	13%
$100,000 to $149,999	15%	16%
$150,000 to $199,999	7%	8%
$200,000 or more	4%	10%
Insurance Status		
Insured	89%	91%
Uninsured	11%	9%
Medicare Status		
Medicare	43%	24%
No Medicare	57%	76%

*What to Notice in *Table 3*:* The demographic profile of the final sample generally matched that of the U.S. general population, as reported in the 2020 U.S. Census. The sample did deviate from the Census regarding Medicare status but not overall insurance status. The larger percentage of respondents on Medicare in our study is likely due to the oversampling of older adults

*Notes*: The U.S. Census percentages for Geographic Area are from the 2010 Census; rural population information was not reported in the 2020 Census

^a^ The U.S. Census asks respondents their “sex” and gives two mutually exclusive response options: Male or Female

Our survey is generalizable only to English-speaking adults in the U.S. who have the requisite internet access and digital literacy to take an online survey. Within that group, it is unlikely that language or literacy barriers affected participation or understanding of the survey. In recruitment, Verasight limited the respondent pool to individuals who were both aged 18 years or older and who could read English. Furthermore, when asked about the highest level of education they had completed, less than 4% of the sample indicated that this was “Some high school or less.” In contrast, approximately 34% of respondents graduated high school or earned a GED, while the remaining 62% had completed at least some college.

### Statistical Analysis

Data were analyzed using STATA (version 15.1) [57]. We used OLS linear regression to test the effect of each predictor variable on the outcome variables.^5^ Each model included the following eight predictors: *Black*, *Hispanic*, *rural*, *age*, *income*, *MRI experience*, *research skepticism*, and *perception of racial bias*. Our analyses utilized survey weights provided by Verasight to correct for sampling biases and non-response in the survey panel, and to ensure that our results were representative with respect to the Current Population Survey benchmarks mentioned previously in the Recruitment section. All analyses were conducted using the “svy” prefix command in STATA to apply the survey weights as appropriate. Tables detailing the results of the regression analyses as well as the percentages of respondents who selected each response option for each outcome of interest can be found in Online Resource 2 (Tables S1-S8).

## Results

### Public Willingness to Participate in Portable MRI Research Is High

**Fig. 1:**
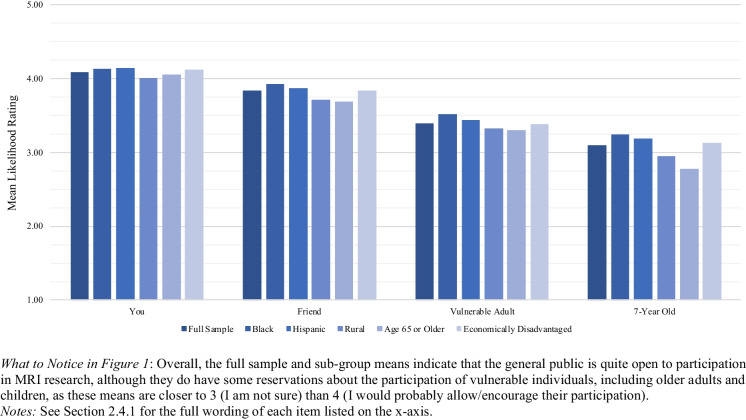
Likelihood of Participating in Portable MRI Research: Mean Ratings by Demographic Sub-Group

Overall, 80% of our respondents would probably or certainly participate in a pMRI study, and 69% would probably or certainly encourage a friend to participate (Table S1**)**. These willingness percentages dropped when we asked about probably or certainly allowing an older or vulnerable adult (55%) or 7-year-old child (42%) to participate. Even for vulnerable adults and children, however, most respondents would not necessarily refuse. Only 26% of respondents would refuse to allow an older or vulnerable adult to participate, and 34% would refuse to allow a 7-year-old child to participate (Table S1). As shown in Fig. 1, there were no substantial differences between the oversampled demographic sub-groups of interest on any of the four outcome variables. When asked about themselves, a friend, or a vulnerable adult, all sub-group means were above the midpoint of the response scale; only when asked about a 7-year-old child’s participation did we see any sub-group means fall below the scale midpoint (for Rural, Older Adults, and Economically Disadvantaged; Table 4).

**Table 4:**
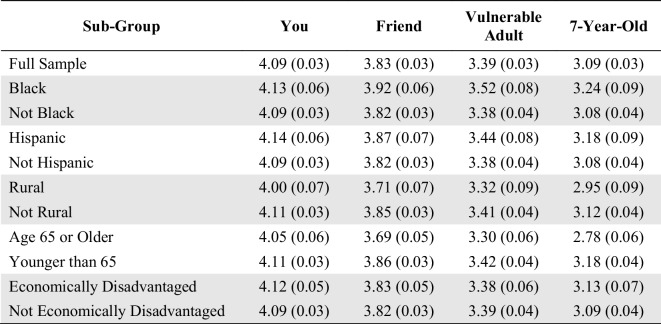
Means (SEs) for Likelihood of Participating in Portable MRI Research Items by Demographic Sub-Group

*What to Notice in *Table 4: Overall, sub-group differences – while potentially significant (see Table S2) – were not particularly meaningful, as most sub-group means were above 4 (I would probably participate) regarding their own participation and above the midpoint of 3 (I am not sure) for the remaining three participation scenarios. Just two means fall below the scale midpoint: Rural (2.95) and Older (2.78) respondents when asked about allowing a 7-year-old child to participate

*Notes*: Means are followed by standard errors in parentheses

Multiple linear regression analysis (Table S2**)** confirmed that demographic characteristics were generally unrelated to respondents’ willingness to participate in pMRI research: Of the 32 effects examined, just 8 were statistically significant (*p* <.05). The few statistically significant relationships were not practically significant given the close similarities in sub-group means (Table 4). For example, *Black* was a significant positive predictor of the *Friend* outcome, suggesting that, compared to non-Black respondents, Black respondents were more likely to encourage a friend to participate in pMRI research. However, the difference in means was merely 0.10 (3.92 for Black respondents and 3.82 for non-Black respondents), suggesting that despite the significance of the predictor variable, both sub-groups were similarly likely to encourage a friend to participate.

**Fig. 2:**
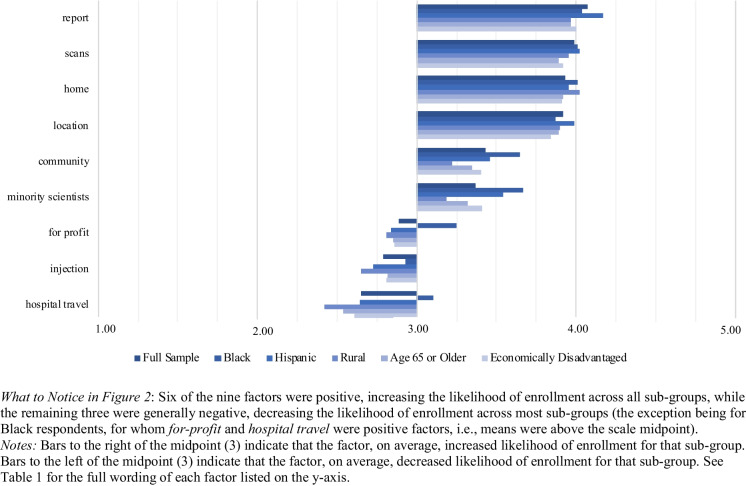
Factors that Might Increase or Decrease the Likelihood of Enrolling in Portable MRI Research: Mean Likelihood of Enrollment Ratings

We next explored nine factors that might increase or decrease the likelihood of an individual deciding to participate in pMRI research (Fig. 2; response frequencies for the full sample are reported in Table S3). Here we asked only about an individual’s own decision to participate, not about a friend, vulnerable adult, or child. Respondents reported that they would be *more* likely to participate in pMRI research when:

- They receive a free MRI brain health report.
- They receive their MRI brain images.
- The research can be completed in a van outside their home.
- The research is at a convenient location nearby.
- The research team consults with members of the respondent’s community.
- The research is to be conducted by a research team that included racial or ethnic minority scientists.

Most respondents reported that they would be *less* likely to enroll in pMRI research when:

- The research is to be conducted by researchers from a private, for-profit company.
- The research requires receiving an injection of a contrast material.
- The research requires travel to and from a hospital.

Multiple linear regression analysis using the weighted data was used to analyze the effect of predictor variables on these nine factors (Table S4). Regression analysis revealed that the most consistent predictor was *research skepticism*: as respondents’ *research skepticism* increased, their likelihood of enrollment tended to decrease, no matter which influential factor was considered.

Among Black respondents, there were two exceptions to this overall pattern. When asked on a scale of 1–5 (with 3 being neutral) whether they would be more or less likely to enroll, the average response of Black respondents was 3.25 if the research was to be conducted by researchers from a private, for-profit company, and 3.10 if the research required travel to and from a hospital. For non-Black respondents, however, the average was 2.84 if the research was to be conducted by researchers from a private, for-profit company, and 2.59 if the research required travel to and from a hospital. Thus, there was a statistically significant difference between Black and non-Black respondents on the impact of these two factors on likelihood of enrolling, though the average response of Black participants was still close to neutral (3). Further investigation is warranted to understand these differences, as they are not explained by the data we collected.

### Respondents Generally Rated Potential pMRI Research Benefits as Highly Important and Potential pMRI Research Harms as Not Important

#### Respondents Rated Many Benefits from Participating in Portable MRI Research as Important

**Fig. 3:**
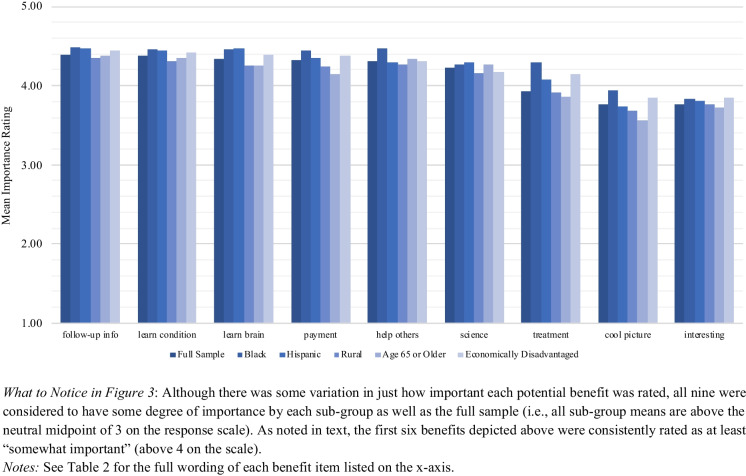
Perceived Importance of Potential Benefits: Mean Importance Ratings by Demographic Sub-Group

Respondents were asked to rate potential benefits from their own participation in pMRI research. All nine potential benefits presented to respondents were rated as important, with all sub-group means above the midpoint of the response scale (Fig. 3; response frequencies for the full sample are reported in Table S5). Respondents perceived the following benefits as most important (i.e., all sub-group means were above 4 (Somewhat important)):

- Receiving follow-up information on the research.
- Learning more about their medical conditions that need attention.
- Learning more about their brain health.
- Receiving financial compensation for research participation.
- Helping others and providing benefit to future patients.
- Contributing to scientific progress.

Multiple linear regression analysis using the weighted data was employed to analyze the effect of each predictor variable on the perceived importance of the nine potential benefits (Table S6). These results, combined with an examination of the sub-group means (Fig. 3), suggest that the different demographic sub-groups rate these benefits similarly in terms of their importance. The means for the demographic sub-groups did not differ significantly from the overall full sample mean. Regression results suggested that there were no substantively meaningful differences in benefits ratings between sub-groups.^6^

One clear pattern is that Black respondents rated six of the benefits as more important than non-Black respondents: receiving study results; learning about their medical conditions; learning more about their brain health; desiring to help future patients; contributing to scientific progress; and accessing medical treatment. This suggests that although all potential benefits were important to all respondents, these six were especially important to Black respondents.

#### Respondents Generally Rated Concerns about Participating in Portable MRI Research as Not Important

**Fig. 4:**
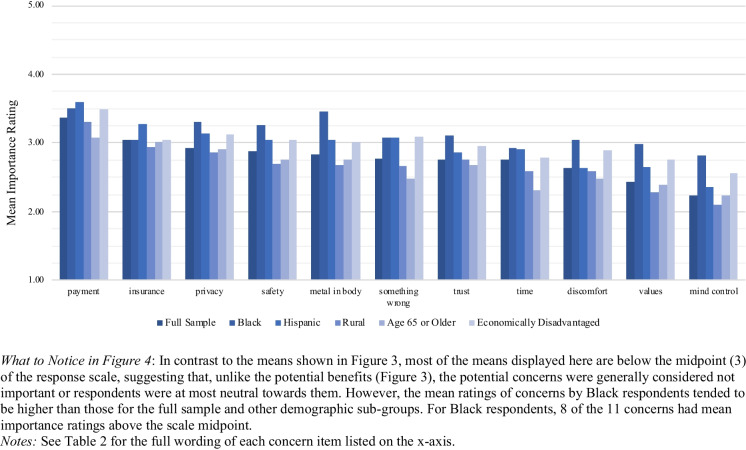
Perceived Importance of Potential Concerns: Mean Importance Ratings by Demographic Sub-Group

Respondents were asked to rate potential concerns about their own participation in pMRI research. In contrast to potential benefits, which were all rated as important, respondents generally did not rate potential concerns as important, with one clear exception (Fig. 4; response frequencies for the full sample are reported in Table S7). The one concern for which the full sample and all sub-group means were above the neutral midpoint of the response scale was the concern that there would not be enough payment for time and travel. The possibility that insurance companies might use research brain scans to raise participants’ insurance rates was rated as the second-most important concern, but below the neutral midpoint.

All other concerns – aside from these two – were not, on average, rated as important by the full sample. That is, the average response on our 1–5 importance scale did not rise above the midpoint of 3. The midpoint 3 represents neither unimportant nor important on the response scale. We note that the mean ratings of concerns by Black respondents tended to be higher than those for the full sample and other demographic sub-groups. For Black respondents, although 8 of the 11 concerns had mean importance ratings at or above the scale midpoint (Fig. 4), none of these ratings reached the value of 4 (corresponding to “somewhat important”). Our data therefore suggest both that Black respondents perceived several concerns as more important than did non-Black respondents, and at the same time that neither group saw the concerns as rising to the level of “somewhat important.” Nevertheless, additional research is needed to explain these response patterns.

Multiple linear regression analysis using the weighted data (Table S8) generally revealed a lack of sub-group differences in rating the importance of concerns. Most of the demographic predictors had inconsistent and small effects on the perceived importance of potential concerns. We did find some significant effects for the *Black* predictor, reflecting the higher sub-group means for Black respondents seen in Fig. 4. Compared to non-Black respondents, Black respondents were, on average, more concerned about three issues: being scanned with metal or electronic devices in their body, the research team not respecting their cultural and community values, and researchers using the brain scan for mind control. However, the means for the latter two concerns were below the scale midpoint, suggesting that although Black respondents rated these concerns as more important than non-Black respondents, they did not necessarily view them as important. These differences are difficult to interpret based on our data and warrant further research.

### Skepticism About Research Reduces Willingness to Participate and Heightens Concerns

We further investigated the factors that drive an individual’s willingness to participate in pMRI research. We examined the effects of several predictor variables on respondents’ own willingness to participate via regression analyses. We found that the higher that respondents rated the following three concerns, the less willing they were to participate in pMRI research: “Brain scan will not be safe,” “Brain Scan would be too uncomfortable/I am claustrophobic,” and “Researchers cannot be trusted/not sure of their motivations.” In addition, we examined the effect of *research skepticism*, our two-item composite variable that assesses the extent to which respondents think medical researchers downplay the dangers associated with participation in medical research and cover up their mistakes (see Sect."Factors to Explain pMRI Attitudes"). The higher respondents scored on this measure (i.e., the more skeptical they were), the less willing they were to participate in pMRI research themselves. We then examined whether *research skepticism* had a similar effect on respondents’ willingness to encourage a friend or allow a child or vulnerable adult to participate. Those who scored higher on *research skepticism* were less likely to allow a 7-year-old child to participate and less likely to encourage a friend to participate, but there was no effect of *research skepticism* on willingness to allow an older or vulnerable adult to participate.

Looking next at the relationship between *research skepticism* and assessment of benefits and concerns, we found that compared to less skeptical respondents, more skeptical respondents generally rated most benefits as less important and rated all concerns as more important. Additionally, respondents’ *perception of racial bias* in medical research was positively correlated with their assessment of concerns associated with pMRI research participation. Specifically, respondents who had a higher *perception of racial bias* rated several concerns as more important, including the researchers not understanding or respecting participants’ cultural and community values.

### Comparing Female vs. Male Attitudes Toward pMRI Research

The literature on portable MRI posits that this new technology could facilitate increased neuroimaging research participation in underrepresented groups such as Black, Hispanic, rural, economically disadvantaged, and older participants [58–60]. By contrast, although sex bias remains a prominent concern in non-human animal research [61], and neuroimaging studies are beginning to explore gender as well as sex differences [62], males and females are generally both well represented in contemporary human neuroimaging research [63]. Our survey was not designed to address sex or gender disparities, and we did not expect to find major differences between females and males in attitudes toward pMRI research. To explore whether this was the case, we conducted additional regression analyses that added sex as a predictor variable. This variable (female) was coded such that 0 = male and 1 = female. Respondents who selected “Other” in response to our demographic question were not included in these analyses, as only 19 respondents chose this category. The regression results from these additional analyses are reported in the Supplemental Materials (Online Resource 2, Tables S9-S12).

Adding the female variable to our original regression model does not change our substantive findings and conclusions. We found that female respondents were somewhat less willing than male respondents to allow a vulnerable adult or 7-year-old child to participate in pMRI research (Table S9). However, for the vulnerable adult item, the means for both female (*M* = 3.32) and male (*M* = 3.47) respondents were above the midpoint of the scale, suggesting hesitation about allowing participation rather than outright refusal by either group. For the 7-year-old child item, the mean for female participants (*M* = 2.98) was nearly at the midpoint of the response scale.

We also found that female respondents rated six of the nine benefits as more important than did male respondents: receiving study results/follow-up information; learning about their medical conditions; learning more about their brain health; desiring to help future patients; contributing to scientific progress; and accessing medical treatment (Table S11). While these means for male and female respondents are different, those differences are small (Table S11). For both males and females, all but one of the means on these six items were between 4 (Somewhat important) and 5 (Very important). This suggests that respondents, regardless of sex, found these benefits important.

## Discussion

### Need for ELSI Guidance

Our results strongly suggest that if pMRI research opportunities expand, a large percentage of those in the U.S. would participate, as 80% of our survey respondents would probably or certainly participate, 69% would recommend a friend participate, 55% would allow an older or vulnerable adult to participate, and 42% would allow a 7-year-old child to participate (Table S1). This high interest in pMRI research – with some hesitancy concerning such research in young children – confirms the importance of developing ELSI guidance now, before pMRI research rapidly expands. The need for ELSI guidance is all the more important because pMRI is so new that the public has yet to encounter the technology to experience directly the ways in which research with pMRI differs from traditional MRI research. Thus, it is not surprising that the respondents in our survey generally did not rate as important many of the ELSI concerns presented to them. However, the need for ethical protections in research is not limited to problems the public perceives and researchers are not allowed to conduct research simply because respondents agree; researchers must also meet standards set by federal regulations and other guidelines addressing additional problems that experts have identified.

In separate work, our project’s Working Group has identified 15 core pMRI research ELSI issues, recommended solutions, and suggested strategies for implementation [3]. Those recommendations cover a range of issues, including: ensuring that research personnel are sufficiently trained; careful design and oversight of research; guaranteeing the safety of research participants and others in the scanning environment; engagement of diverse participants and communities; minimizing the therapeutic misconception; cautious and transparent use of artificial intelligence algorithms to acquire and analyze MRI data; ensuring data privacy and security; managing return of results and incidental findings (IFs); and ensuring research participant data access and control.

In considering why respondents were generally so willing to participate in pMRI research, one plausible explanation is that the public is familiar with and comfortable participating in fixed MRI research, and that they view pMRI research similarly. In our pilot testing, we ran a survey that randomly assigned participants to a question about participation in fixed MRI brain research (scanning in “a magnetic resonance imaging (MRI) machine … at the major hospital or University closest to you”), versus pMRI research (scanning “in a small, portable magnetic resonance imaging (MRI) machine. … in a convenient location near your home such as a community center.”). We found no significant difference in willingness to participate between the two groups. While we were not able to carry out this same experimental design in the final survey due to resource constraints, the pilot data are consistent with an interpretation that participants either see little difference between portable MRI and fixed MRI, or see differences between the technologies but do not consider the differences germane to benefits and concerns. Because our survey took steps to clarify distinctions between pMRI and fixed MRI – including providing a vignette and visual illustration – our respondents were probably answering the survey questions with the image and description of *portable* MRI in mind. But our pilot data that found no difference between fixed MRI and pMRI responses suggest that perhaps respondents see pMRI as just a smaller version of traditional, fixed MRI – not fully understanding the technical differences, including those that lead to lower quality images from pMRI machines.

The details of how pMRI and fixed MRI differ depend on the type of pMRI being utilized. pMRI is not a single technology but a suite of “highly portable and accessible brain MRI technologies, which vary in field strength, spatial resolution, temporal resolution, intended use, cost, and ease of use” [3]. Given that our respondents indicated that receiving a brain health report or copies of their brain scan would increase their likelihood of participating in pMRI research, one of the important differences concerns scanner field strength—and the resulting quality of the scan and utility in clinical care. Some pMRI machines are “low-field” (0.01 < 0.1T) or “ultra-low field” (ULF, < 0.01T), and at present it remains uncertain how these LF and ULF scanners will be used in clinical care [60, 64]. Moreover, diagnostic scanning in clinical care (unlike research pMRI) typically involves selecting a scanning protocol and sequence (with or without contrast media) to target the suspected brain pathology and yield a diagnosis. It is thus important for potential participants to know that participating in pMRI research may not generate the equivalent of a clinical grade scan. An implication is that research teams should make sure they understand the capabilities and limitations of the pMRI device they are using, and explain this to potential participants during the informed consent process to avoid the therapeutic misconception. The therapeutic misconception “is a widely recognized problem in informed consent that occurs when subjects consent to participate in clinical research because of the belief that they will receive the same individually focused treatment that they would receive in a nonresearch clinical context” [65].

Elsewhere we have written more extensively on the potential for the therapeutic misconception to emerge in pMRI research [3, 66]. In research conducted “in remote settings with populations unfamiliar with MRI or facing barriers to clinical access [participants] may easily misconstrue neuroimaging research for clinical care.” To mitigate this concern, researchers should address community and individual needs related to MRI to avoid a mismatch between the researchers’ viewpoints as scientists, and the participants’ viewpoints as individuals who may have medical needs [67]. As we have recommended previously, the “core of the solution is likely to be improved communication, leading to improved understanding by the participant” [3].

### Building Trust with Local Communities

One of the great promises of pMRI is its ability to facilitate MRI research in remote and underserved settings. But this geographical mobility of pMRI could also encourage extractive research in which research teams arrive in a community to scan, acquire MRI data, and then leave without sustained local engagement [2]. The survey data reported here confirms our concern that lack of trust in the research team could hamper participation in pMRI research. Our statistical analysis found that the more important respondents rated the concern “Researchers cannot be trusted/not sure of their motivations,” the less willing they were to participate in pMRI research. Similarly, the more skeptical respondents were of medical research – as measured by the *research skepticism* composite variable – the less likely they were to allow a 7-year-old child to participate or encourage a friend to participate. This finding supports our argument in related work that the first step in field-based pMRI research is to establish community trust [14].

Our data also show that respondents who agree with the statement that “medical researchers act differently toward minority subjects than toward white subjects” are less willing to participate in pMRI research. This further supports the importance of building trust with research participants, especially those from groups that have historically been underrepresented in neuroimaging research.

Our survey data also found that for Black and Hispanic respondents – compared to respondents in neither sub-group – a more racially or ethnically diverse research team significantly increased their willingness to participate in pMRI research. On this item, the mean for Black respondents was 3.67 compared to 3.33 for non-Black respondents. Similarly, the mean for Hispanic respondents was 3.54 compared to 3.34 for non-Hispanic respondents. This finding reinforces the importance of a recommendation we have made in related work, that portable MRI “research teams should be composed of individuals from diverse backgrounds” [3]. But the complexities of participant recruitment caution against an overly simplistic approach. As research has shown, “researcher honesty and shared values” are also likely to be important [68].

Relatedly, when we asked respondents, “If the research was led by a research team that consulted with members of your community before conducting the study, would you be more or less likely to participate in the research study?”, the mean for Black respondents was 3.65 whereas the mean for non-Black respondents was 3.40. This difference was statistically significant, though the magnitude of the difference is small. Although Black respondents had slightly higher means on this question, those means did not reach the “more likely to enroll” value of 4. Still, this highlights the potential importance of community engagement for this sub-group. Further work is needed to better understand how respondents understood the term “your community,” but the finding is consistent with the Working Group’s recommendations that consultation with the community before research begins should be prioritized [14].

Consultation with the community would also serve a related function: helping potential participants understand the limitations and potential concerns with pMRI research, as well as the research team’s plan for addressing those concerns. For example, the pMRI research team could address the potential that the pMRI scan might reveal an incidental finding, offering a clinical grade scan at a different location. The team could also address how the research project will ensure a pathway to timely follow-up care for those participants with IFs or research results warranting clinical referral [3, 69].

Practitioners in community engagement have developed a variety of concrete strategies that researchers can deploy to build trust with and work with community members. These include: community engagement studios [70], community benefits agreements [71], and community advisory boards [72]. In related work we have discussed how such strategies can be integrated into the lifecycle of a portable neuroimaging research study, and provided researchers with relevant resources [14]. Hemley and colleagues have pioneered the use of a “targeted stakeholder group (TSG) to develop a theory of change (ToC)” [73]. The TSG approach is a “structured method to obtain input from stakeholders that enhances general research design, conduct, and dissemination.” The stakeholder group meets monthly with the research team with a “focus on building models to resolve long-term issues around research engagement and bidirectional benefit.” The ToC method may be promising for expanding participation in brain research.

### Delivering on pMRI Research Benefits and Addressing pMRI Research Concerns

Our survey respondents rated as important many potential benefits of pMRI research. For example, benefits such as providing a brain health report, identification of medical conditions needing attention, and access to medical treatment were seen as important benefits across all demographic sub-groups. This strongly suggests that investigators and funders should consider providing these benefits. At the very least, researchers will need to consult with the community of prospective participants to create an acceptable plan for managing IFs and offering a referral pathway to follow-up clinical care [69]. At the same time, researchers should take steps to ensure that the community and prospective participants are not over-estimating the benefits that pMRI research can provide. Starting with initial community engagement efforts and the informed consent process, and continuing throughout the lifecycle of the research project, researchers should clarify for participants what benefits can and cannot be expected from their participation. pMRI investigators need to identify, communicate, and address ELSI issues. In related work, we recommended that “before carrying out the research, researchers conducting portable MRI research should become familiar with the ELSI issues identified in this article, and investigators designing research should partner with the local communities in which research will occur” [3]. This includes minimizing the therapeutic misconception by ensuring that potential participants are aware of the difference between the proposed research and clinical care.

The most important concern for our survey respondents was that they would not receive sufficient payment for the time and travel required by research participation. These ratings may reflect multiple factors, such as respondents not having a baseline for what “proper” compensation is or holding the view that research participants are generally undercompensated no matter what the study. Whatever the underlying reason for this concern, the research team should ensure, through community engagement before the research begins, that adequate compensation is provided to all research participants [14]. Additionally, if sub-groups have particular concerns about pMRI research those concerns need to be addressed, perhaps through deeper community engagement and the provision of additional information about pMRI research. For example, Black respondents in our study rated “Metal or electronic device in my body” (*M* = 3.45) and the research team not respecting their cultural and community values (*M* = 2.98) as more important concerns than did non-Black respondents (*M* = 2.75 and *M* = 2.36, respectively). However, all of these group means are below the scale midpoint, indicating that, on average, they were not seen as important by either Black or non-Black respondents. Nevertheless, the potential for sub-group differences suggests that pMRI research teams need to engage communities with potential sub-group participants to develop strategies to effectively ascertain and address concerns those communities and individuals may have.

### Limitations

The results presented in this article are from a single survey, featuring one vignette and visually depicting one type of pMRI device. Our results are generalizable only to English speakers in the U.S. and are subject to debates discussed above about the potential limitations of probabilistic vs. non-probabilistic approaches to survey methodology. Additional work is required to better understand public sentiment across a wider range of pMRI devices, research contexts, non-English speakers, and countries.

Our findings raise several new questions for future research. Additional research could help to uncover whether the generally low importance that respondents assigned to potential concerns was due to genuine lack of concern, or instead the result of the technologies being so new that few participants had direct experience with the technology as yet. Re-administration of the survey as pMRI moves further along the translational arc might produce different results. Additional media coverage of portable neuroimaging over time may also affect public opinion. Future research could also investigate the differences we observed in some responses by Black participants, including why the for-profit and hospital travel factors increased the likelihood of participation for only Black respondents (Fig. 2).

One of the potential benefits of pMRI is its ability to facilitate MRI research in rural and lower-resource communities. But the rural respondents and economically disadvantaged respondents in our survey did not offer significantly different responses on participation, benefits ratings, or concerns ratings. One limitation that might explain these null results is that the self-reported *Rural* variable included individuals who were not sufficiently geographically remote to see great value in pMRI that can travel to them. In a separate question we asked respondents, “If you had to travel to a major hospital, how long would it take you to travel there?” In answering this question, even 72% of rural respondents live within 1 hour of a hospital.

As with all studies that elicit only stated preferences, those preferences may not predict actual behavior [74, 75]. Following established methods [76], future research could examine actual decisions about whether to enroll in pMRI studies, including possible disparities in enrollment rates across demographic sub-groups. Real world studies would allow participants to see the specific pMRI equipment being used, speak with the research team, and gain more information with which to make their decision. Factors not captured here, such as the quality of the engagement and characteristics of the recruitment team, might play a significant role in actual enrollment.

Finally, the importance of our results depends on how widely pMRI is used for research, now and in the future. Portable MRI is already playing a critical role in clinical neuroscience research. For instance, studies are examining if pMRI can be used to improve diagnosis of hydrocephalus [77], treatment of patients in the ICU [78], and assessment of brain injury [79]. In addition, pMRI may play a role in more basic neuroscience research that seeks to examine the brain-behavior relationship for more complex behavioral and psychological phenomena. In the realm of this brain-behavior research, introduction of pMRI comes at a time where the field is debating the value of cross-sectional MRI research [80], with increasing recognition of the limits of small-N studies [81]. Yet, in a study critiquing the value of small-N cross-sectional studies, Marek et al. conclude that “our prospects for linking neuroimaging markers to complex human behaviours are better than ever,” if researchers adopt research paradigms that feature larger sample sizes, within-participant designs, and interventional designs [80]. Portable MRI may prove most valuable in well-designed studies enrolling the new populations that only highly portable technologies can reach.

## Conclusion

Our analysis of the first nationally representative survey on pMRI research finds high public willingness to participate in pMRI research studies. Notably, our data do not reveal major differences in willingness to participate across key demographic sub-groups, suggesting that pMRI may enable a new era of MRI research featuring community-based projects and robust participation across locations and demographic groups.

The public’s interest in pMRI research seems driven by their perception of many potential benefits and few potential concerns attending pMRI research. If these benefits are not realized, if concerns emerge that the public is not anticipating, and if the ELSI issues we identify here are not addressed, then the vision of offering greater access to pMRI research and building more representative data sets may not be realized. With careful attention to ELSI issues and partnership with participant communities, pMRI appears poised to open a new, more inclusive chapter in brain research.

## Supplementary Information

Below is the link to the electronic supplementary material.

Supplementary file1 (DOCX 144 KB)

Supplementary file2 (DOCX 232 KB)

## Data Availability

Replication data for this study is available at: https://dataverse.harvard.edu/dataverse/portableMRI/.

## Footnotes

1 Response options varied slightly depending on the question. Options for the “friend” question ranged from (1) I would certainly not encourage participation, to (5) I would certainly encourage participation. Options for the “vulnerable adult” and “7-year-old” questions ranged from (1) I would certainly refuse their participation, to (5) I would certainly allow their participation.

2 Although our survey offered the three choices listed in text in asking respondents to self-identify their gender, we recognize that there is a wide spectrum of gender identities [82].

3 In setting the population benchmarks, Verasight used sex and not gender because at present the Current Population Survey data allow for benchmarking on sex but not gender. An extensive literature addresses the distinctions between sex and gender [82, 83]. How to appropriately incorporate gender into population benchmarking in survey research is an area of active investigation [84].

4 The Office of the Assistant Secretary for Planning and Evaluation (Department of Health and Human Services) provides three sets of poverty guidelines based on location (i.e., 48 contiguous states and the District of Columbia, Alaska, and Hawaii). A different poverty guideline is provided based on the number of persons in the household. Respondents were first classified by location based on their self-reported zip code. Next, using location and self-reported household number, respondents were labelled Economically Disadvantaged (or not) based on their reported income bracket.

5 As a robustness check, alternative model specifications utilizing ordered logit and ordered probit regression were explored. These alternative model specifications did not change the substantive results reported here.

6 Although *age* and *income* were statistically significant in several models explaining the benefits outcomes, the magnitude of these effects was so small as to have little practical significance. As seen in Fig. 3, sub-group means were quite similar – indicating that all respondents perceived these benefits as important, despite differences in age and income.
