# Supplementary material for: Revolutionizing Brain Research Using Portable MRI in Field Settings: Public Perspectives on the Ethical and Legal Challenges
Source: Neuroethics. 2025 Jul 26;18(2):36. doi: 10.1007/s12152-025-09606-4 (PMC12296799; doi:10.1007/s12152-025-09606-4)
Supplement: Supplementary file 1 — Supplementary file1 (DOCX 144 KB) [file 12152_2025_9606_MOESM1_ESM.docx]

**Online Resource 2**

**Table S1**

*Likelihood of Participating in Portable MRI Research: Full Sample Means, SDs, and Percentage of Respondents who Selected Each Response Option.*

| **Participant** | **Mean** | **SD** | **Certainly Refuse (1)** | **Probably Refuse (2)** | **Not Sure (3)** | **Probably Participate (4)** | **Certainly Participate (5)** |
| --- | --- | --- | --- | --- | --- | --- | --- |
| You | 4.09 | 0.03 | 3.1% | 5.3% | 12.7% | 37.0% | 42.0% |
| Friend | 3.83 | 0.03 | 2.0% | 8.4% | 21.0% | 42.4% | 26.3% |
| Older or Vulnerable Adult | 3.39 | 0.03 | 8.7% | 17.8% | 19.0% | 34.7% | 19.8% |
| 7-Year Old Child | 3.09 | 0.03 | 13.5% | 20.9% | 23.3% | 27.2% | 15.1% |

**Table S2**

*Regression Table: Likelihood of Participating in Portable MRI Research*

| **Predictors** |  | **You** | **Friend** | **Vulnerable Adult** | **7-Year Old** |
| --- | --- | --- | --- | --- | --- |
|  |  |  |  |  |  |
| Black | Coefficient | 0.127 | 0.147* | 0.203* | 0.175 |
|  | Std. Error | (0.077) | (0.071) | (0.098) | (0.104) |
|  | *p*-value | .099 | .039 | .038 | .094 |
|  |  |  |  |  |  |
| Hispanic | Coefficient | 0.081 | 0.038 | 0.102 | 0.036 |
|  | Std. Error | (0.076) | (0.078) | (0.100) | (0.100) |
|  | *p*-value | .286 | .630 | .309 | .719 |
|  |  |  |  |  |  |
| Rural | Coefficient | -0.099 | -0.092 | -0.067 | -0.092 |
|  | Std. Error | (0.074) | (0.071) | (0.097) | (0.094) |
|  | *p*-value | .178 | .195 | .490 | .328 |
|  |  |  |  |  |  |
| Age | Coefficient | -0.003 | -0.005** | -0.001 | -0.008*** |
|  | Std. Error | (0.002) | (0.002) | (0.002) | (0.002) |
|  | *p*-value | .143 | .001 | .651 | .000 |
|  |  |  |  |  |  |
| Income | Coefficient | -0.003 | 0.007 | 0.010 | 0.004 |
|  | Std. Error | (0.006) | (0.007) | (0.008) | (0.008) |
|  | *p*-value | .619 | .264 | .231 | .622 |
|  |  |  |  |  |  |
| MRI Experience | Coefficient | 0.130* | 0.046 | 0.065 | -0.050 |
|  | Std. Error | (0.058) | (0.054) | (0.076) | (0.076) |
|  | *p*-value | .025 | .395 | .388 | .510 |
|  |  |  |  |  |  |
| Research Skepticism | Coefficient | -0.223*** | -0.184*** | -0.073 | -0.232*** |
|  | Std. Error | (0.028) | (0.028) | (0.038) | (0.036) |
|  | *p*-value | .000 | .000 | .054 | .000 |
|  |  |  |  |  |  |
| Perception of Racial Bias | Coefficient | -0.026 | -0.016 | -0.037 | 0.016 |
|  | Std. Error | (0.028) | (0.027) | (0.037) | (0.035) |
|  | *p*-value | .348 | .563 | .319 | .653 |
|  |  |  |  |  |  |
| Constant | Coefficient | 4.872*** | 4.570*** | 3.609*** | 4.083*** |
|  | Std. Error | (0.140) | (0.132) | (0.185) | (0.177) |
|  | *p*-value | .000 | .000 | .000 | .000 |
|  |  |  |  |  |  |
| Observations |  | 1,994 | 1,994 | 1,994 | 1,994 |
| R-squared |  | .070 | .055 | .011 | .051 |

| **Predictors** |  | **You** | **Friend** | **Vulnerable Adult** | **7-Year Old** |
| --- | --- | --- | --- | --- | --- |
|  |  |  |  |  |  |
| Black | Coefficient | 0.127 | 0.147* | 0.203* | 0.175 |
|  | Std. Error | (0.077) | (0.071) | (0.098) | (0.104) |
|  | *p*-value | 0.099 | 0.039 | 0.038 | 0.094 |
|  |  |  |  |  |  |
| Hispanic | Coefficient | 0.081 | 0.038 | 0.102 | 0.036 |
|  | Std. Error | (0.076) | (0.078) | (0.100) | (0.100) |
|  | *p*-value | 0.286 | 0.630 | 0.309 | 0.719 |
|  |  |  |  |  |  |
| Rural | Coefficient | -0.099 | -0.092 | -0.067 | -0.092 |
|  | Std. Error | (0.074) | (0.071) | (0.097) | (0.094) |
|  | *p*-value | 0.178 | 0.195 | 0.490 | 0.328 |
|  |  |  |  |  |  |
| Age | Coefficient | -0.003 | -0.005** | -0.001 | -0.008*** |
|  | Std. Error | (0.002) | (0.002) | (0.002) | (0.002) |
|  | *p*-value | 0.143 | 0.001 | 0.651 | 0.000 |
|  |  |  |  |  |  |
| Income | Coefficient | -0.003 | 0.007 | 0.010 | 0.004 |
|  | Std. Error | (0.006) | (0.007) | (0.008) | (0.008) |
|  | *p*-value | 0.619 | 0.264 | 0.231 | 0.622 |
|  |  |  |  |  |  |
| MRI Experience | Coefficient | 0.130* | 0.046 | 0.065 | -0.050 |
|  | Std. Error | (0.058) | (0.054) | (0.076) | (0.076) |
|  | *p*-value | 0.025 | 0.395 | 0.388 | 0.510 |
|  |  |  |  |  |  |
| Research Skepticism | Coefficient | -0.223*** | -0.184*** | -0.073 | -0.232*** |
|  | Std. Error | (0.028) | (0.028) | (0.038) | (0.036) |
|  | *p*-value | 0.000 | 0.000 | 0.054 | 0.000 |
|  |  |  |  |  |  |
| Pereption of Racial Bias | Coefficient | -0.026 | -0.016 | -0.037 | 0.016 |
|  | Std. Error | (0.028) | (0.027) | (0.037) | (0.035) |
|  | *p*-value | 0.348 | 0.563 | 0.319 | 0.653 |
|  |  |  |  |  |  |
| Constant | Coefficient | 4.872*** | 4.570*** | 3.609*** | 4.083*** |
|  | Std. Error | (0.140) | (0.132) | (0.185) | (0.177) |
|  | *p*-value | 0.000 | 0.000 | 0.000 | 0.000 |
|  |  |  |  |  |  |
| Observations |  | 1,994 | 1,994 | 1,994 | 1,994 |
| R-squared |  | 0.070 | 0.055 | 0.011 | 0.051 |

*What to Notice in Table S2*: The most consistent predictor of these outcomes was research skepticism, with three negative effects. Overall, and in line with the means presented in Figure 1, demographic and other background characteristics did not seem to play a large role in respondents’ likelihood of participating in portable MRI research.

*Notes:* Significant effects are marked as follows: ****p*<.001, ***p*<.01, and **p*<.05.

**Table S3**

*Factors that Might Increase or Decrease the Likelihood of Enrolling in Portable MRI Research: Full Sample Means, SDs, and Percentage of Respondents who Selected Each Response Option.*

| **Influential Factor** | **Mean** | | **SD** | | **Much Less Likely to Enroll (1)** | | **Less Likely to Enroll (2)** | | **Neutral (3)** | | **More Likely to Enroll (4)** | | **Much More Likely to Enroll (5)** | |  |
| --- | --- | --- | --- | --- | --- | --- | --- | --- | --- | --- | --- | --- | --- | --- | --- |
| Report | 4.07 | | 0.03 | | 2.4% | | 2.4% | | 17.0% | | 42.3% | | 35.9% | |  |
| Scans | 3.99 | | 0.02 | | 1.7% | | 2.4% | | 23.0% | | 40.8% | | 32.1% | |  |
| Home | 3.93 | | 0.03 | | 4.0% | | 6.7% | | 18.6% | | 33.3% | | 37.4% | |  |
| Location | 3.92 | | 0.03 | | 2.4% | | 4.6% | | 21.1% | | 42.7% | | 29.3% | |  |
| Community | 3.43 | | 0.02 | | 2.9% | | 5.9% | | 49.7% | | 28.3% | | 13.1% | |  |
| Minority Scientists | 3.37 | | 0.02 | | 3.2% | | 3.3% | | 60.8% | | 18.6% | | 14.2% | |  |
| For Profit | 2.89 | | 0.03 | | 9.9% | | 20.8% | | 47.7% | | 14.0% | | 7.6% | |  |
| Injection | 2.79 | | 0.03 | | 16.6% | | 20.0% | | 39.7% | | 14.7% | | 9.0% | |  |
| Hospital Travel | 2.65 | | 0.03 | | 16.6% | | 29.2% | | 34.0% | | 13.2% | | 7.0% | |  |
|  | |  | |  | |  | |  | |  | |  | |  | |

**Table S4**

*Regression Table for Final Model: Influential Factors Battery*

| **Predictors** |  | **report** | **scans** | **home** | **location** | **community** | **minority scientists** | **for profit** | **injection** | **hospital travel** |
| --- | --- | --- | --- | --- | --- | --- | --- | --- | --- | --- |
|  |  |  |  |  |  |  |  |  |  |  |
| Black | Coefficient | 0.032 | 0.081 | 0.181* | 0.009 | 0.204** | 0.327*** | 0.420*** | 0.199* | 0.530*** |
|  | Std. Error | (0.067) | (0.064) | (0.080) | (0.072) | (0.071) | (0.069) | (0.082) | (0.096) | (0.092) |
|  | *p*-value | .626 | .201 | .024 | .897 | .004 | .000 | .000 | .038 | .000 |
|  |  |  |  |  |  |  |  |  |  |  |
| Hispanic | Coefficient | 0.111 | 0.034 | 0.098 | 0.106 | -0.008 | 0.207** | -0.011 | -0.009 | 0.060 |
|  | Std. Error | (0.075) | (0.072) | (0.090) | (0.077) | (0.072) | (0.073) | (0.089) | (0.090) | (0.089) |
|  | *p*-value | .137 | .633 | .274 | .171 | .909 | .004 | .905 | .923 | .502 |
|  |  |  |  |  |  |  |  |  |  |  |
| Rural | Coefficient | -0.061 | -0.018 | 0.130 | 0.020 | -0.196** | -0.137** | -0.071 | -0.172* | -0.220** |
|  | Std. Error | (0.062) | (0.062) | (0.082) | (0.068) | (0.063) | (0.049) | (0.065) | (0.079) | (0.072) |
|  | *p*-value | .324 | .776 | .112 | .771 | .002 | .005 | .275 | .029 | .002 |
|  |  |  |  |  |  |  |  |  |  |  |
| Age | Coefficient | -0.004** | -0.005** | -0.000 | -0.003 | -0.005** | -0.002 | -0.000 | 0.003 | -0.001 |
|  | Std. Error | (0.002) | (0.002) | (0.002) | (0.002) | (0.002) | (0.001) | (0.002) | (0.002) | (0.002) |
|  | *p*-value | .005 | .001 | .866 | .055 | .002 | .259 | .963 | .082 | .666 |
|  |  |  |  |  |  |  |  |  |  |  |
| Income | Coefficient | 0.020** | 0.019** | 0.014 | 0.017** | 0.010 | 0.000 | 0.003 | -0.002 | 0.015* |
|  | Std. Error | (0.007) | (0.006) | (0.007) | (0.007) | (0.006) | (0.006) | (0.007) | (0.008) | (0.007) |
|  | *p*-value | .002 | .003 | .069 | .009 | .117 | .942 | .626 | .813 | .041 |
|  |  |  |  |  |  |  |  |  |  |  |
| MRI Experience | Coefficient | 0.052 | 0.062 | 0.118 | 0.046 | -0.028 | -0.007 | -0.086 | 0.069 | 0.063 |
|  | Std. Error | (0.052) | (0.051) | (0.065) | (0.054) | (0.050) | (0.049) | (0.060) | (0.067) | (0.066) |
|  | *p*-value | .318 | .225 | .069 | .395 | .584 | .879 | .151 | .300 | .347 |
|  |  |  |  |  |  |  |  |  |  |  |
| Research Skepticism | Coefficient | -0.181*** | -0.128*** | -0.145*** | -0.145*** | -0.094*** | -0.160*** | 0.032 | -0.168*** | -0.101** |
|  | Std. Error | (0.027) | (0.025) | (0.031) | (0.028) | (0.027) | (0.025) | (0.032) | (0.033) | (0.032) |
|  | *p*-value | .000 | .000 | .000 | .000 | .000 | .000 | .322 | .000 | .002 |
|  |  |  |  |  |  |  |  |  |  |  |
| Perception of Racial Bias | Coefficient | 0.013 | -0.033 | 0.004 | -0.004 | 0.049 | 0.117*** | -0.064* | 0.025 | 0.013 |
|  | Std. Error | (0.026) | (0.026) | (0.031) | (0.027) | (0.025) | (0.024) | (0.031) | (0.032) | (0.030) |
|  | *p*-value | .617 | .202 | .902 | .890 | .052 | .000 | .037 | .422 | .671 |
|  |  |  |  |  |  |  |  |  |  |  |
| Constant | Coefficient | 4.560*** | 4.492*** | 4.128*** | 4.310*** | 3.742*** | 3.549*** | 2.961*** | 3.038*** | 2.756*** |
|  | Std. Error | (0.130) | (0.126) | (0.161) | (0.133) | (0.129) | (0.120) | (0.145) | (0.156) | (0.144) |
|  | *p*-value | .000 | .000 | .000 | .000 | .000 | .000 | .000 | .000 | .000 |
|  |  |  |  |  |  |  |  |  |  |  |
| Observations |  | 1,994 | 1,994 | 1,994 | 1,994 | 1,994 | 1,994 | 1,994 | 1,994 | 1,994 |
| R-squared |  | .059 | .044 | .028 | .037 | .041 | .067 | .022 | .032 | .040 |

*What to Notice in Table S4*: With the exception of *MRI experience*, each predictor had at least one significant effect across the nine outcomes, with the most consistent predictor being *research skepticism*. Perhaps the most practically significant results occurred for the *minority scientists* factor, as five of the eight predictor variables significantly predicted this outcome.

*Notes:* Significant effects are marked as follows: ****p*<.001, ***p*<.01, and **p*<.05.

**Table S5**

*Perceived Importance of Potential Benefits: Full Sample Means, SDs, and Percentage of Respondents who Selected Each Response Option.*

| **Benefit** | **Mean** | **SD** | **Not Important (1)** | **Somewhat Not Important (2)** | **Neither (3)** | **Somewhat Important (4)** | **Very important (5)** |
| --- | --- | --- | --- | --- | --- | --- | --- |
| Follow-Up Info | 4.39 | 0.02 | 1.2% | 2.2% | 7.2% | 35.0% | 54.4% |
| Learn Condition | 4.37 | 0.02 | 1.8% | 2.0% | 8.5% | 32.4% | 55.2% |
| Learn Brain Health | 4.34 | 0.02 | 1.5% | 1.8% | 8.1% | 37.9% | 50.7% |
| Payment | 4.32 | 0.02 | 1.5% | 3.6% | 6.5% | 38.4% | 50.1% |
| Help Others | 4.31 | 0.02 | 0.9% | 2.1% | 7.7% | 44.2% | 45.1% |
| Science | 4.22 | 0.02 | 1.1% | 2.5% | 11.1% | 44.0% | 41.4% |
| Treatment | 3.93 | 0.03 | 3.5% | 4.3% | 20.5% | 38.8% | 33.0% |
| Cool Picture | 3.77 | 0.03 | 6.1% | 6.4% | 21.8% | 36.3% | 29.5% |
| Interesting | 3.76 | 0.03 | 5.4% | 5.7% | 21.6% | 42.1% | 25.2% |

**Table S6**

*Regression Table for Final Model: Importance of Potential Benefits Items*

| **Predictors** |  | **follow-up information** | **learn condition** | **learn brain** | **payment** | **help others** | **science** | **treatment** | **cool picture** | **interesting** |
| --- | --- | --- | --- | --- | --- | --- | --- | --- | --- | --- |
|  |  |  |  |  |  |  |  |  |  |  |
| Black | Coefficient | 0.164** | 0.161* | 0.195** | 0.124 | 0.215*** | 0.151* | 0.414*** | 0.155 | 0.103 |
|  | Std. Error | (0.058) | (0.064) | (0.060) | (0.065) | (0.053) | (0.059) | (0.069) | (0.081) | (0.082) |
|  | *p*-value | .005 | .012 | .001 | .055 | .000 | .010 | .000 | .057 | .211 |
|  |  |  |  |  |  |  |  |  |  |  |
| Hispanic | Coefficient | 0.150* | 0.143 | 0.207*** | -0.017 | 0.049 | 0.157** | 0.215** | -0.085 | 0.064 |
|  | Std. Error | (0.061) | (0.073) | (0.063) | (0.069) | (0.065) | (0.061) | (0.076) | (0.093) | (0.088) |
|  | *p*-value | .014 | .050 | .001 | .809 | .451 | .010 | .005 | .361 | .464 |
|  |  |  |  |  |  |  |  |  |  |  |
| Rural | Coefficient | -0.039 | -0.068 | -0.086 | -0.087 | -0.041 | -0.040 | 0.025 | -0.076 | 0.034 |
|  | Std. Error | (0.066) | (0.063) | (0.059) | (0.069) | (0.053) | (0.055) | (0.069) | (0.077) | (0.074) |
|  | *p*-value | .552 | .278 | .145 | .210 | .442 | .463 | .713 | .324 | .649 |
|  |  |  |  |  |  |  |  |  |  |  |
| Age | Coefficient | 0.001 | -0.000 | -0.002 | -0.006*** | 0.001 | 0.002 | -0.000 | -0.005** | -0.001 |
|  | Std. Error | (0.001) | (0.002) | (0.001) | (0.002) | (0.001) | (0.001) | (0.002) | (0.002) | (0.002) |
|  | *p*-value | .570 | .819 | .284 | .000 | .380 | .171 | .853 | .005 | .729 |
|  |  |  |  |  |  |  |  |  |  |  |
| Income | Coefficient | -0.006 | -0.003 | -0.000 | -0.013* | 0.000 | 0.007 | -0.024*** | -0.017* | -0.011 |
|  | Std. Error | (0.005) | (0.006) | (0.006) | (0.006) | (0.005) | (0.006) | (0.006) | (0.007) | (0.007) |
|  | *p*-value | .274 | .565 | .981 | .023 | .938 | .199 | .000 | .022 | .122 |
|  |  |  |  |  |  |  |  |  |  |  |
| MRI Experience | Coefficient | 0.040 | 0.050 | 0.104* | 0.049 | 0.088* | 0.054 | 0.019 | -0.070 | -0.022 |
|  | Std. Error | (0.045) | (0.050) | (0.046) | (0.052) | (0.045) | (0.046) | (0.057) | (0.065) | (0.065) |
|  | *p*-value | .379 | .317 | .023 | .341 | .049 | .250 | .739 | .279 | .736 |
|  |  |  |  |  |  |  |  |  |  |  |
| Research Skepticism | Coefficient | -0.034 | -0.054* | -0.084*** | 0.027 | -0.090*** | -0.177*** | -0.002 | -0.119*** | -0.153*** |
|  | Std. Error | (0.023) | (0.026) | (0.025) | (0.024) | (0.021) | (0.021) | (0.028) | (0.033) | (0.033) |
|  | *p*-value | .140 | .039 | .001 | .266 | .000 | .000 | .943 | .000 | .000 |
|  |  |  |  |  |  |  |  |  |  |  |
| Perception of Racial Bias | Coefficient | -0.024 | -0.026 | -0.017 | -0.040 | 0.019 | 0.028 | 0.044 | 0.019 | 0.053 |
|  | Std. Error | (0.021) | (0.023) | (0.025) | (0.025) | (0.022) | (0.022) | (0.027) | (0.031) | (0.031) |
|  | *p*-value | .270 | .271 | .501 | .103 | .396 | .215 | .100 | .552 | .083 |
|  |  |  |  |  |  |  |  |  |  |  |
| Constant | Coefficient | 4.501*** | 4.585*** | 4.607*** | 4.720*** | 4.382*** | 4.444*** | 3.922*** | 4.499*** | 4.161*** |
|  | Std. Error | (0.112) | (0.115) | (0.120) | (0.113) | (0.110) | (0.111) | (0.144) | (0.147) | (0.141) |
|  | *p*-value | .000 | .000 | .000 | .000 | .000 | .000 | .000 | .000 | .000 |
|  |  |  |  |  |  |  |  |  |  |  |
| Observations |  | 1,994 | 1,994 | 1,994 | 1,994 | 1,994 | 1,994 | 1,994 | 1,994 | 1,994 |
| R-squared |  | .012 | .015 | .031 | .021 | .025 | .056 | .035 | .025 | .021 |

*What to Notice in Table S6*: The most consistent predictors of the perceived importance of potential benefits were *Black* and *research skepticism*, with six significant effects each. The effects of additional explanatory variables were significant in some models but not in others. These variables were less consistently significant than those highlighted and, where significant, tended to be of a smaller magnitude.

*Notes:* Significant effects are marked as follows: ****p*<.001, ***p*<.01, and **p*<.05.

**Table S7**

*Perceived Importance of Potential Concerns: Full Sample Means, SDs, and Percentage of Respondents who Selected Each Response Option.*

| **Concern** | **Mean** | **SD** | **Not Important (1)** | **Somewhat Not Important (2)** | **Neither (3)** | **Somewhat Important (4)** | **Very important (5)** |
| --- | --- | --- | --- | --- | --- | --- | --- |
| Payment | 3.36 | 0.03 | 11.7% | 11.7% | 24.1% | 34.6% | 18.0% |
| Insurance | 3.05 | 0.04 | 24.3% | 12.0% | 17.4% | 27.3% | 19.0% |
| Privacy | 2.92 | 0.04 | 26.5% | 14.8% | 18.6% | 20.7% | 19.4% |
| Safety | 2.88 | 0.04 | 28.1% | 14.4% | 17.0% | 22.7% | 17.8% |
| Metal In Body | 2.83 | 0.04 | 33.5% | 9.8% | 15.3% | 22.9% | 18.5% |
| Something Wrong | 2.77 | 0.04 | 27.1% | 16.3% | 20.3% | 25.1% | 11.1% |
| Trust | 2.76 | 0.04 | 26.5% | 16.5% | 24.0% | 19.9% | 13.1% |
| Time | 2.75 | 0.04 | 24.9% | 17.9% | 24.0% | 24.4% | 8.9% |
| Discomfort | 2.64 | 0.04 | 33.8% | 14.0% | 18.0% | 23.2% | 11.0% |
| Values | 2.43 | 0.04 | 37.3% | 14.8% | 25.1% | 12.8% | 10.0% |
| Mind Control | 2.24 | 0.04 | 50.0% | 11.8% | 13.6% | 12.9% | 11.7% |

**Table S8**

*Regression Table for Final Model: Potential Concerns Items*

| **Predictors** |  | **payment** | **insurance** | **privacy** | **safety** | **metal in body** | **something wrong** | **trust** | **time** | **discomfort** | **values** | **mind control** |
| --- | --- | --- | --- | --- | --- | --- | --- | --- | --- | --- | --- | --- |
|  |  |  |  |  |  |  |  |  |  |  |  |  |
| Black | Coefficient | -0.002 | -0.205 | 0.200 | 0.185 | 0.507*** | 0.109 | 0.145 | -0.094 | 0.180 | 0.362*** | 0.415*** |
|  | Std. Error | (0.095) | (0.108) | (0.108) | (0.108) | (0.115) | (0.110) | (0.098) | (0.101) | (0.106) | (0.102) | (0.112) |
|  | *p*-value | .980 | .058 | .063 | .086 | .000 | .322 | .140 | .354 | .091 | .000 | .000 |
|  |  |  |  |  |  |  |  |  |  |  |  |  |
| Hispanic | Coefficient | 0.134 | 0.180 | 0.228* | 0.099 | 0.202 | 0.205* | 0.048 | -0.035 | -0.107 | 0.206* | 0.111 |
|  | Std. Error | (0.092) | (0.111) | (0.108) | (0.107) | (0.120) | (0.100) | (0.097) | (0.096) | (0.114) | (0.100) | (0.101) |
|  | *p*-value | .146 | .104 | .035 | .358 | .093 | .040 | .623 | .715 | .348 | .039 | .271 |
|  |  |  |  |  |  |  |  |  |  |  |  |  |
| Rural | Coefficient | 0.007 | -0.077 | -0.011 | -0.139 | -0.091 | -0.036 | 0.031 | -0.097 | -0.004 | -0.105 | -0.135 |
|  | Std. Error | (0.092) | (0.103) | (0.097) | (0.098) | (0.108) | (0.097) | (0.090) | (0.092) | (0.100) | (0.082) | (0.092) |
|  | *p*-value | .943 | .458 | .905 | .157 | .403 | .711 | .731 | .291 | .965 | .202 | .144 |
|  |  |  |  |  |  |  |  |  |  |  |  |  |
| Age | Coefficient | -0.007** | 0.000 | 0.003 | 0.000 | 0.000 | -0.006** | -0.000 | -0.014*** | -0.002 | 0.002 | 0.005* |
|  | Std. Error | (0.002) | (0.002) | (0.002) | (0.002) | (0.003) | (0.002) | (0.002) | (0.002) | (0.002) | (0.002) | (0.002) |
|  | *p*-value | .002 | .975 | .291 | .961 | .975 | .009 | .874 | .000 | .449 | .295 | .037 |
|  |  |  |  |  |  |  |  |  |  |  |  |  |
| Income | Coefficient | -0.008 | 0.012 | -0.015 | -0.005 | -0.015 | -0.033*** | -0.013 | 0.013 | -0.024** | -0.033*** | -0.032*** |
|  | Std. Error | (0.008) | (0.009) | (0.009) | (0.009) | (0.010) | (0.009) | (0.009) | (0.008) | (0.009) | (0.008) | (0.009) |
|  | *p*-value | .302 | .216 | .101 | .613 | .130 | .000 | .138 | .106 | .006 | .000 | .000 |
|  |  |  |  |  |  |  |  |  |  |  |  |  |
| MRI Experience | Coefficient | -0.162* | -0.073 | -0.100 | -0.279*** | -0.366*** | -0.229** | -0.127 | -0.113 | -0.203* | -0.202** | -0.101 |
|  | Std. Error | (0.071) | (0.084) | (0.079) | (0.083) | (0.090) | (0.078) | (0.075) | (0.075) | (0.082) | (0.076) | (0.076) |
|  | *p*-value | .023 | .385 | .207 | .001 | .000 | .003 | .088 | .129 | .013 | .008 | .188 |
|  |  |  |  |  |  |  |  |  |  |  |  |  |
| Research Skepticism | Coefficient | 0.227*** | 0.292*** | 0.401*** | 0.385*** | 0.369*** | 0.168*** | 0.455*** | 0.219*** | 0.272*** | 0.272*** | 0.370*** |
|  | Std. Error | (0.033) | (0.041) | (0.039) | (0.040) | (0.042) | (0.036) | (0.036) | (0.035) | (0.038) | (0.036) | (0.037) |
|  | *p*-value | .000 | .000 | .000 | .000 | .000 | .000 | .000 | .000 | .000 | .000 | .000 |
|  |  |  |  |  |  |  |  |  |  |  |  |  |
| Perception of Racial Bias | Coefficient | 0.091** | 0.173*** | 0.204*** | 0.152*** | 0.078 | 0.193*** | 0.129*** | 0.140*** | 0.179*** | 0.239*** | 0.169*** |
|  | Std. Error | (0.033) | (0.039) | (0.039) | (0.038) | (0.040) | (0.036) | (0.034) | (0.034) | (0.037) | (0.034) | (0.036) |
|  | *p*-value | .005 | .000 | .000 | .000 | .054 | .000 | .000 | .000 | .000 | .000 | .000 |
|  |  |  |  |  |  |  |  |  |  |  |  |  |
| Constant | Coefficient | 2.902*** | 1.684*** | 1.198*** | 1.526*** | 1.794*** | 2.391*** | 1.253*** | 2.382*** | 1.750*** | 1.198*** | 0.734*** |
|  | Std. Error | (0.169) | (0.206) | (0.192) | (0.200) | (0.213) | (0.196) | (0.181) | (0.179) | (0.198) | (0.185) | (0.189) |
|  | *p*-value | .000 | .000 | .000 | .000 | .000 | .000 | .000 | .000 | .000 | .000 | .000 |
|  |  |  |  |  |  |  |  |  |  |  |  |  |
| Observations |  | 1,994 | 1,994 | 1,994 | 1,994 | 1,994 | 1,994 | 1,994 | 1,994 | 1,994 | 1,994 | 1,994 |
| R-squared |  | .099 | .100 | .171 | .153 | .133 | .117 | .192 | .135 | .118 | .173 | .155 |

*What to Notice in Table S8*: The most consistent predictors of the perceived importance of potential concerns were *research skepticism, perception of racial bias*, and *MRI experience*. The effects of additional explanatory variables were significant in some models but not in others. These variables were less consistently significant than those highlighted and, where significant, tended to be of a smaller magnitude.

*Notes:* Significant effects are marked as follows: ****p*<.001, ***p*<.01, and **p*<.05.

**Table S9**

*Regression Table for Model Including Gender: Likelihood of Participating in Portable MRI Research*

| **Predictors** |  | **You** | **Friend** | **Vulnerable Adult** | **7-Year Old** |
| --- | --- | --- | --- | --- | --- |
|  |  |  |  |  |  |
| Black | Coefficient | 0.135 | 0.145* | 0.189 | 0.183 |
|  | Std. Error | (0.078) | (0.072) | (0.097) | (0.103) |
|  | *p*-value | .083 | .044 | .051 | .074 |
|  |  |  |  |  |  |
| Hispanic | Coefficient | 0.077 | 0.038 | 0.077 | 0.030 |
|  | Std. Error | (0.077) | (0.079) | (0.102) | (0.101) |
|  | *p*-value | .315 | .637 | .451 | .765 |
|  |  |  |  |  |  |
| Rural | Coefficient | -0.088 | -0.093 | -0.088 | -0.080 |
|  | Std. Error | (0.075) | (0.073) | (0.099) | (0.094) |
|  | *p*-value | .240 | .200 | .374 | .395 |
|  |  |  |  |  |  |
| Age | Coefficient | -0.003 | -0.005** | -0.002 | -0.008*** |
|  | Std. Error | (0.002) | (0.002) | (0.002) | (0.002) |
|  | *p*-value | .142 | .001 | .486 | .000 |
|  |  |  |  |  |  |
| Income | Coefficient | -0.004 | 0.007 | 0.007 | -0.003 |
|  | Std. Error | (0.007) | (0.007) | (0.009) | (0.009) |
|  | *p*-value | .501 | .315 | .430 | .769 |
|  |  |  |  |  |  |
| MRI Experience | Coefficient | 0.136* | 0.050 | 0.078 | -0.038 |
|  | Std. Error | (0.058) | (0.055) | (0.076) | (0.076) |
|  | *p*-value | .020 | .362 | .309 | .618 |
|  |  |  |  |  |  |
| Research Skepticism | Coefficient | -0.220*** | -0.183*** | -0.083* | -0.239*** |
|  | Std. Error | (0.029) | (0.028) | (0.038) | (0.036) |
|  | *p*-value | .000 | .000 | .027 | .000 |
|  |  |  |  |  |  |
| Perception of Racial Bias | Coefficient | -0.029 | -0.012 | -0.024 | 0.024 |
|  | Std. Error | (0.028) | (0.027) | (0.037) | (0.035) |
|  | *p*-value | .315 | .664 | .519 | .492 |
|  |  |  |  |  |  |
| Female | Coefficient | -0.050 | -0.032 | -0.154* | -0.253*** |
|  | Std. Error | (0.055) | (0.053) | (0.069) | (0.067) |
|  | *p*-value | .362 | .549 | .026 | .000 |
|  |  |  |  |  |  |
| Constant | Coefficient | 4.898*** | 4.579*** | 3.747*** | 4.266*** |
|  | Std. Error | (0.149) | (0.140) | (0.197) | (0.181) |
|  | *p*-value | .000 | .000 | .000 | .000 |
|  |  |  |  |  |  |
| Observations |  | 1,975 | 1,975 | 1,975 | 1,975 |
| R-squared |  | .069 | .054 | .015 | .060 |

*What to Notice in Table S9*: Even after adding *female* to the model, the most consistent predictor of these outcomes remained *research skepticism*. *Female* had two significant negative effects, indicating that, compared to male respondents, female respondents were less likely to allow a vulnerable adult or 7-year-old child participate in pMRI research.

*Notes:* The variable for gender, *female,* was coded such that 0 = male and 1 = female. Significant effects are marked as follows: ****p*<.001, ***p*<.01, and **p*<.05.

**Table S10**

*Regression Table for Model Including Gender: Influential Factors Battery*

| **Predictors** | **Value** | **report** | **scans** | **home** | **location** | **community** | **minority scientists** | **for profit** | **injection** | **hospital travel** |
| --- | --- | --- | --- | --- | --- | --- | --- | --- | --- | --- |
|  |  |  |  |  |  |  |  |  |  |  |
| Black | Coefficient | 0.041 | 0.080 | 0.166* | 0.008 | 0.213** | 0.359*** | 0.408*** | 0.236* | 0.536*** |
|  | Std. Error | (0.068) | (0.064) | (0.079) | (0.072) | (0.071) | (0.068) | (0.082) | (0.093) | (0.091) |
|  | *p*-value | .545 | .212 | .035 | .911 | .003 | .000 | .000 | .011 | .000 |
|  |  |  |  |  |  |  |  |  |  |  |
| Hispanic | Coefficient | 0.109 | 0.045 | 0.045 | 0.076 | -0.017 | 0.205** | -0.023 | -0.007 | 0.057 |
|  | Std. Error | (0.075) | (0.073) | (0.089) | (0.078) | (0.073) | (0.073) | (0.090) | (0.091) | (0.091) |
|  | *p*-value | .148 | .537 | .615 | .331 | .816 | .005 | .800 | .943 | .527 |
|  |  |  |  |  |  |  |  |  |  |  |
| Rural | Coefficient | -0.069 | -0.025 | 0.160* | 0.007 | -0.199** | -0.149** | -0.058 | -0.165* | -0.203** |
|  | Std. Error | (0.062) | (0.063) | (0.075) | (0.069) | (0.063) | (0.048) | (0.065) | (0.078) | (0.073) |
|  | *p*-value | .273 | .691 | .033 | .914 | .002 | .002 | .372 | .035 | .006 |
|  |  |  |  |  |  |  |  |  |  |  |
| Age | Coefficient | -0.004* | -0.004** | -0.002 | -0.003* | -0.005** | -0.001 | -0.001 | 0.003 | -0.001 |
|  | Std. Error | (0.002) | (0.002) | (0.002) | (0.002) | (0.002) | (0.001) | (0.002) | (0.002) | (0.002) |
|  | *p*-value | .013 | .004 | .411 | .044 | .002 | .517 | .579 | .130 | .527 |
|  |  |  |  |  |  |  |  |  |  |  |
| Income | Coefficient | 0.023*** | 0.019** | 0.013 | 0.018** | 0.008 | -0.001 | -0.001 | -0.012 | 0.009 |
|  | Std. Error | (0.007) | (0.007) | (0.008) | (0.007) | (0.006) | (0.007) | (0.007) | (0.008) | (0.008) |
|  | *p*-value | .001 | .004 | .087 | .008 | .218 | .835 | .929 | .129 | .215 |
|  |  |  |  |  |  |  |  |  |  |  |
| MRI Experience | Coefficient | 0.054 | 0.057 | 0.115 | 0.054 | -0.035 | -0.001 | -0.079 | 0.088 | 0.065 |
|  | Std. Error | (0.052) | (0.052) | (0.064) | (0.055) | (0.050) | (0.049) | (0.060) | (0.067) | (0.067) |
|  | *p*-value | .301 | .266 | .073 | .321 | .487 | .988 | .192 | .190 | .334 |
|  |  |  |  |  |  |  |  |  |  |  |
| Research Skepticism | Coefficient | -0.178*** | -0.126*** | -0.149*** | -0.150*** | -0.090*** | -0.155*** | 0.022 | -0.171*** | -0.104** |
|  | Std. Error | (0.027) | (0.026) | (0.031) | (0.028) | (0.027) | (0.025) | (0.032) | (0.033) | (0.032) |
|  | *p*-value | .000 | .000 | .000 | .000 | .001 | .000 | .504 | .000 | .001 |
|  |  |  |  |  |  |  |  |  |  |  |
| Perception of Racial Bias | Coefficient | 0.006 | -0.036 | 0.008 | -0.003 | 0.043 | 0.105*** | -0.050 | 0.028 | 0.016 |
|  | Std. Error | (0.027) | (0.026) | (0.030) | (0.028) | (0.025) | (0.024) | (0.031) | (0.031) | (0.030) |
|  | *p*-value | .833 | .165 | .780 | .924 | .088 | .000 | .107 | .372 | .588 |
|  |  |  |  |  |  |  |  |  |  |  |
| Female | Coefficient | 0.049 | 0.031 | -0.135* | -0.058 | -0.088 | -0.079 | -0.137* | -0.293*** | -0.192** |
|  | Std. Error | (0.051) | (0.050) | (0.058) | (0.052) | (0.047) | (0.046) | (0.055) | (0.060) | (0.060) |
|  | *p*-value | .329 | .531 | .021 | .272 | .061 | .083 | .012 | .000 | .001 |
|  |  |  |  |  |  |  |  |  |  |  |
| Constant | Coefficient | 4.503*** | 4.453*** | 4.274*** | 4.355*** | 3.824*** | 3.572*** | 3.105*** | 3.272*** | 2.917*** |
|  | Std. Error | (0.139) | (0.135) | (0.165) | (0.141) | (0.133) | (0.126) | (0.156) | (0.160) | (0.151) |
|  | *p*-value | .000 | .000 | .000 | .000 | .000 | .000 | .000 | .000 | .000 |
|  |  |  |  |  |  |  |  |  |  |  |
| Observations |  | 1,975 | 1,975 | 1,975 | 1,975 | 1,975 | 1,975 | 1,975 | 1,975 | 1,975 |
| R-squared |  | .059 | .044 | .033 | .040 | .044 | .066 | .024 | .047 | .045 |

*What to Notice in Table S10*: As with the original model (Table S4), with the exception of *MRI experience*, each predictor had at least one significant effect across the nine outcomes. Even after adding *female* to the model, *research skepticism* remained the most consistent predictor. *Female* had four significant negative effects.

*Notes:* The variable for gender, *female,* was coded such that 0 = male and 1 = female. Significant effects are marked as follows: ****p*<.001, ***p*<.01, and **p*<.05.

**Table S11**

*Regression Table for Model Including Gender: Importance of Potential Benefits Items*

| **Predictors** | **Value** | **follow-up information** | **learn condition** | **learn brain** | **payment** | **help others** | **science** | **treatment** | **cool picture** | **interesting** |
| --- | --- | --- | --- | --- | --- | --- | --- | --- | --- | --- |
|  |  |  |  |  |  |  |  |  |  |  |
| Black | Coefficient | 0.147* | 0.158* | 0.186** | 0.115 | 0.213*** | 0.155** | 0.400*** | 0.163* | 0.114 |
|  | Std. Error | (0.057) | (0.063) | (0.059) | (0.065) | (0.053) | (0.059) | (0.069) | (0.082) | (0.083) |
|  | *p*-value | .010 | .013 | .002 | .080 | .000 | .009 | .000 | .048 | .169 |
|  |  |  |  |  |  |  |  |  |  |  |
| Hispanic | Coefficient | 0.154** | 0.159* | 0.221*** | -0.009 | 0.075 | 0.183** | 0.222** | -0.069 | 0.074 |
|  | Std. Error | (0.059) | (0.072) | (0.062) | (0.071) | (0.066) | (0.061) | (0.076) | (0.094) | (0.088) |
|  | *p*-value | .010 | .028 | .000 | .899 | .258 | .003 | .004 | .463 | .403 |
|  |  |  |  |  |  |  |  |  |  |  |
| Rural | Coefficient | -0.024 | -0.092 | -0.098 | -0.093 | -0.058 | -0.055 | 0.020 | -0.082 | 0.022 |
|  | Std. Error | (0.060) | (0.061) | (0.059) | (0.070) | (0.053) | (0.054) | (0.069) | (0.079) | (0.076) |
|  | *p*-value | .694 | .133 | .096 | .185 | .273 | .314 | .774 | .302 | .771 |
|  |  |  |  |  |  |  |  |  |  |  |
| Age | Coefficient | 0.001 | 0.001 | -0.001 | -0.006*** | 0.002 | 0.003 | -0.000 | -0.005* | -0.000 |
|  | Std. Error | (0.001) | (0.002) | (0.001) | (0.002) | (0.001) | (0.001) | (0.002) | (0.002) | (0.002) |
|  | *p*-value | .514 | .708 | .514 | .000 | .104 | .077 | .998 | .011 | .919 |
|  |  |  |  |  |  |  |  |  |  |  |
| Income | Coefficient | -0.000 | 0.003 | 0.005 | -0.012* | 0.006 | 0.009 | -0.019** | -0.016* | -0.012 |
|  | Std. Error | (0.005) | (0.006) | (0.006) | (0.006) | (0.006) | (0.006) | (0.007) | (0.008) | (0.007) |
|  | *p*-value | .955 | .664 | .342 | .034 | .299 | .104 | .004 | .033 | .102 |
|  |  |  |  |  |  |  |  |  |  |  |
| MRI Experience | Coefficient | 0.025 | 0.045 | 0.094* | 0.041 | 0.084 | 0.053 | 0.005 | -0.068 | -0.015 |
|  | Std. Error | (0.045) | (0.050) | (0.045) | (0.052) | (0.045) | (0.047) | (0.057) | (0.065) | (0.065) |
|  | *p*-value | .576 | .365 | .040 | .432 | .061 | .261 | .925 | .300 | .819 |
|  |  |  |  |  |  |  |  |  |  |  |
| Research Skepticism | Coefficient | -0.026 | -0.047 | -0.079** | 0.030 | -0.082*** | -0.174*** | 0.002 | -0.112*** | -0.145*** |
|  | Std. Error | (0.023) | (0.026) | (0.025) | (0.025) | (0.021) | (0.021) | (0.028) | (0.034) | (0.033) |
|  | *p*-value | .259 | .065 | .002 | .223 | .000 | .000 | .934 | .001 | .000 |
|  |  |  |  |  |  |  |  |  |  |  |
| Perception of Racial Bias | Coefficient | -0.031 | -0.036 | -0.024 | -0.040 | 0.013 | 0.025 | 0.038 | 0.012 | 0.048 |
|  | Std. Error | (0.022) | (0.023) | (0.024) | (0.025) | (0.022) | (0.022) | (0.027) | (0.032) | (0.031) |
|  | *p*-value | .148 | .125 | .332 | .110 | .562 | .274 | .161 | .700 | .117 |
|  |  |  |  |  |  |  |  |  |  |  |
| Female | Coefficient | 0.193*** | 0.227*** | 0.206*** | 0.050 | 0.224*** | 0.137** | 0.179*** | 0.049 | -0.016 |
|  | Std. Error | (0.043) | (0.046) | (0.045) | (0.048) | (0.042) | (0.043) | (0.054) | (0.062) | (0.059) |
|  | *p*-value | .000 | .000 | .000 | .291 | .000 | .001 | .001 | .429 | .782 |
|  |  |  |  |  |  |  |  |  |  |  |
| Constant | Coefficient | 4.361*** | 4.379*** | 4.438*** | 4.683*** | 4.162*** | 4.323*** | 3.791*** | 4.441*** | 4.136*** |
|  | Std. Error | (0.115) | (0.125) | (0.128) | (0.118) | (0.116) | (0.118) | (0.150) | (0.158) | (0.150) |
|  | *p*-value | .000 | .000 | .000 | .000 | .000 | .000 | .000 | .000 | .000 |
|  |  |  |  |  |  |  |  |  |  |  |
| Observations |  | 1,975 | 1,975 | 1,975 | 1,975 | 1,975 | 1,975 | 1,975 | 1,975 | 1,975 |
| R-squared |  | .025 | .033 | .046 | .022 | .047 | .065 | .040 | .024 | .020 |

*What to Notice in Table S11*: As in the original analyses (Table S6), the most consistent predictors of the perceived importance of potential benefits were *Black* and *research skepticism*. *Female* was also a consistent predictor, with six significant positive effects.

*Notes:* The variable for gender, *female,* was coded such that 0 = male and 1 = female. Significant effects are marked as follows: ****p*<.001, ***p*<.01, and **p*<.05.

**Table S12**

*Regression Table for Model Including Gender: Potential Concerns Items*

| **Predictors** | **Value** | **payment** | **insurance** | **privacy** | **safety** | **metal in body** | **something wrong** | **trust** | **time** | **discomfort** | **values** | **mind control** |
| --- | --- | --- | --- | --- | --- | --- | --- | --- | --- | --- | --- | --- |
|  |  |  |  |  |  |  |  |  |  |  |  |  |
| Black | Coefficient | -0.018 | -0.210 | 0.187 | 0.171 | 0.496*** | 0.065 | 0.128 | -0.113 | 0.176 | 0.361*** | 0.394*** |
|  | Std. Error | (0.095) | (0.109) | (0.108) | (0.109) | (0.115) | (0.112) | (0.099) | (0.102) | (0.108) | (0.103) | (0.113) |
|  | *p*-value | .853 | .054 | .084 | .117 | .000 | .560 | .195 | .265 | .104 | .000 | .000 |
|  |  |  |  |  |  |  |  |  |  |  |  |  |
| Hispanic | Coefficient | 0.105 | 0.166 | 0.202 | 0.103 | 0.227 | 0.207* | 0.027 | -0.064 | -0.063 | 0.189 | 0.108 |
|  | Std. Error | (0.093) | (0.112) | (0.109) | (0.106) | (0.121) | (0.099) | (0.098) | (0.097) | (0.113) | (0.101) | (0.102) |
|  | *p*-value | .262 | .140 | .065 | .332 | .060 | .037 | .782 | .509 | .577 | .061 | .291 |
|  |  |  |  |  |  |  |  |  |  |  |  |  |
| Rural | Coefficient | 0.032 | -0.052 | -0.002 | -0.092 | -0.046 | -0.029 | 0.057 | -0.077 | -0.016 | -0.087 | -0.107 |
|  | Std. Error | (0.088) | (0.102) | (0.095) | (0.094) | (0.107) | (0.092) | (0.088) | (0.089) | (0.097) | (0.082) | (0.092) |
|  | *p*-value | .716 | .612 | .986 | .325 | .667 | .753 | .518 | .391 | .867 | .292 | .246 |
|  |  |  |  |  |  |  |  |  |  |  |  |  |
| Age | Coefficient | -0.008*** | -0.000 | 0.002 | -0.000 | -0.000 | -0.006** | -0.001 | -0.015*** | -0.001 | 0.002 | 0.004 |
|  | Std. Error | (0.002) | (0.002) | (0.002) | (0.002) | (0.003) | (0.002) | (0.002) | (0.002) | (0.002) | (0.002) | (0.002) |
|  | *p*-value | .000 | .931 | .414 | .997 | .856 | .005 | .736 | .000 | .549 | .324 | .084 |
|  |  |  |  |  |  |  |  |  |  |  |  |  |
| Income | Coefficient | -0.007 | 0.012 | -0.016 | -0.000 | -0.015 | -0.025** | -0.010 | 0.014 | -0.023* | -0.033*** | -0.033*** |
|  | Std. Error | (0.008) | (0.010) | (0.010) | (0.010) | (0.010) | (0.009) | (0.009) | (0.009) | (0.009) | (0.009) | (0.009) |
|  | *p*-value | .399 | .205 | .094 | .980 | .127 | .007 | .279 | .111 | .010 | .000 | .000 |
|  |  |  |  |  |  |  |  |  |  |  |  |  |
| MRI Experience | Coefficient | -0.175* | -0.084 | -0.117 | -0.297*** | -0.383*** | -0.249** | -0.140 | -0.124 | -0.203* | -0.217** | -0.112 |
|  | Std. Error | (0.072) | (0.085) | (0.080) | (0.083) | (0.091) | (0.078) | (0.075) | (0.075) | (0.082) | (0.076) | (0.077) |
|  | *p*-value | .015 | .320 | .143 | .000 | .000 | .001 | .064 | .099 | .014 | .004 | .144 |
|  |  |  |  |  |  |  |  |  |  |  |  |  |
| Research Skepticism | Coefficient | 0.226*** | 0.296*** | 0.403*** | 0.394*** | 0.366*** | 0.165*** | 0.455*** | 0.214*** | 0.264*** | 0.280*** | 0.367*** |
|  | Std. Error | (0.034) | (0.041) | (0.039) | (0.041) | (0.042) | (0.036) | (0.036) | (0.035) | (0.038) | (0.036) | (0.037) |
|  | *p*-value | .000 | .000 | .000 | .000 | .000 | .000 | .000 | .000 | .000 | .000 | .000 |
|  |  |  |  |  |  |  |  |  |  |  |  |  |
| Perception of Racial Bias | Coefficient | 0.089** | 0.167*** | 0.200*** | 0.147*** | 0.079 | 0.195*** | 0.126*** | 0.142*** | 0.185*** | 0.231*** | 0.176*** |
|  | Std. Error | (0.033) | (0.040) | (0.039) | (0.038) | (0.040) | (0.036) | (0.035) | (0.034) | (0.037) | (0.034) | (0.036) |
|  | *p*-value | .007 | .000 | .000 | .000 | .050 | .000 | .000 | .000 | .000 | .000 | .000 |
|  |  |  |  |  |  |  |  |  |  |  |  |  |
| Female | Coefficient | 0.001 | 0.009 | -0.090 | 0.165* | 0.082 | 0.290*** | 0.031 | -0.035 | 0.155* | -0.024 | -0.031 |
|  | Std. Error | (0.066) | (0.078) | (0.074) | (0.075) | (0.081) | (0.071) | (0.069) | (0.067) | (0.073) | (0.067) | (0.072) |
|  | *p*-value | .991 | .913 | .225 | .027 | .310 | .000 | .656 | .603 | .035 | .716 | .661 |
|  |  |  |  |  |  |  |  |  |  |  |  |  |
| Constant | Coefficient | 2.976*** | 1.700*** | 1.302*** | 1.404*** | 1.798*** | 2.227*** | 1.252*** | 2.475*** | 1.647*** | 1.222*** | 0.801*** |
|  | Std. Error | (0.174) | (0.219) | (0.199) | (0.209) | (0.224) | (0.202) | (0.185) | (0.185) | (0.204) | (0.192) | (0.196) |
|  | *p*-value | .000 | .000 | .000 | .000 | .000 | .000 | .000 | .000 | .000 | .000 | .000 |
|  |  |  |  |  |  |  |  |  |  |  |  |  |
| Observations |  | 1,975 | 1,975 | 1,975 | 1,975 | 1,975 | 1,975 | 1,975 | 1,975 | 1,975 | 1,975 | 1,975 |
| R-squared |  | .102 | .099 | .171 | .159 | .135 | .127 | .192 | .139 | .121 | .172 | .156 |

*What to Notice in Table S12*: As in the original analyses, the most consistent predictors of the perceived importance of potential concerns were *research skepticism, perception of racial bias*, and *MRI experience*. *Female* had three positive significant effects.

*Notes:* The variable for gender, *female,* was coded such that 0 = male and 1 = female. Significant effects are marked as follows: ****p*<.001, ***p*<.01, and **p*<.05.
