# Supplementary material for: Revolutionizing Brain Research Using Portable MRI in Field Settings: Public Perspectives on the Ethical and Legal Challenges
Source: Neuroethics. 2025 Jul 26;18(2):36. doi: 10.1007/s12152-025-09606-4 (PMC12296799; doi:10.1007/s12152-025-09606-4)
Supplement: Supplementary file 2 — Supplementary file2 (DOCX 232 KB) [file 12152_2025_9606_MOESM2_ESM.docx]

**Online Resource 1**

**Full Text of General Public Survey**

Note: The formatting of the text and questions appears differently on the Qualtrics platform. But the text is identical to what is presented here.

Text in red is internal and is NOT presented to participants.

**INFORMED CONSENT**

**INFORMATION SHEET FOR RESEARCH**

Participation in Brain Science Research

XXX IRB Study ID: XXX

We invite you to join a research study focused on people’s willingness to participate in brain science research. You were selected as a possible participant because you meet the Verasight qualification criteria. We ask that you read this form and ask any questions you may have before agreeing to be in the study.

**Procedures:** If you agree to be in this study, we would ask you to do the following things:

· Read and evaluate a series of short scenarios and questions that follow those scenarios.

· Answer a series of background questions. These will include questions about your age, gender, race, and education.

· At no point will you be asked any question that could personally identify you.

· We estimate that the survey will take you about 8 minutes to complete.

**Confidentiality:** The records of this study will be kept private. In any report we might publish, we will not include any information that will make it possible to identify a subject. Research records will be stored securely and only researchers will have access to the records.

**Voluntary Nature of the Study:** Participation in this study is voluntary. Your decision whether or not to participate will not affect your current or future relations with [redacted]. If you decide to participate, you are free to not answer any question or withdraw at any time without affecting those relationships.

**Will I be compensated for my participation?** If you agree to take part in this research study, you will be compensated by Verasight for your time and effort.

**Contacts and Questions:** The researchers conducting this study are: [redacted]

This research has been reviewed and approved by an IRB within the Human Research Protections Program (HRPP). To share feedback privately with the HRPP about your research experience, call XXX. You are encouraged to contact the HRPP if:

- Your questions, concerns, or complaints are not being answered by the research team.
- You cannot reach the research team.
- You want to talk to someone besides the research team.
- You have questions about your rights as a research participant.
- You want to get information or provide input about this research.

Having now read this information about the study, **you can choose whether to click the button below to verify that you give your informed consent to participation in this survey.**

**VIGNETTE**

Imagine that a research team at a local university is studying adults and kids to learn how human brain structure changes with age. The research studies include a brain scanning session in a small, portable magnetic resonance imaging (MRI) machine.

The MRI scan will take 30 minutes. Including time before and after the brain scan, the total time required for the study is 2 hours. The research team will follow all COVID-19 safety protocols.

There are no direct benefits to participants, but all participants will be paid $150.

The picture below depicts what the portable MRI machine could look like.


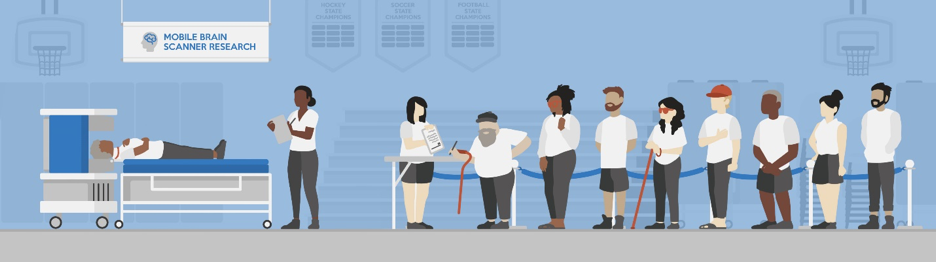


The next four questions are randomly presented, to account for possible order effects.

**[Q1a] PARTICIPATION QUESTION – YOU**

QUESTION: How likely would you be to participate in the research study?

Response options:

1. I would certainly refuse to participate
2. I would probably refuse to participate
3. I am not sure
4. I would probably participate
5. I would certainly participate

**[Q1b] PARTICIPATION QUESTION – CHILD**

QUESTION: If you had a 7 year old child, how likely would you be to allow your child to participate in the research study?

Response options:

1. I would certainly refuse their participation
2. I would probably refuse their participation
3. I am not sure
4. I would probably allow their participation
5. I would certainly allow their participation

**[Q1c] PARTICIPATION QUESTION – OLDER ADULT**

QUESTION: If you were the decision-maker for an older adult who could not make complicated decisions for themselves, how likely would you be to allow this older or vulnerable adult to participate in the research study?

Response options:

1. I would certainly refuse their participation
2. I would probably refuse their participation
3. I am not sure
4. I would probably allow their participation
5. I would certainly allow their participation

**[Q1d] PARTICIPATION QUESTION – FRIEND**

QUESTION: How likely would you be to encourage a close friend to participate in the research study?

Response options:

1. I would certainly not encourage participation
2. I would probably not encourage participation
3. I am not sure
4. I would probably encourage participation
5. I would certainly encourage participation

**[Q2] FOLLOW UP: CONVENIENT LOCATION**

QUESTION: If the research team were able to **set up the portable MRI in a convenient location near your home** such as a community center, would this make you more or less likely to participate in the research study?

Response options:

1. Much less likely to enroll
2. Less likely to enroll
3. Neutral
4. More likely to enroll
5. Much more likely to enroll

**[Q3] FOLLOW UP: MRI IN A VAN TO YOUR HOME**

QUESTION: If the research team were able to **put the MRI machine in a van and come to your home to do the scan without requiring you to travel**, would this make you more or less likely to participate in the research study?

Response options:

1. Much less likely to enroll
2. Less likely to enroll
3. Neutral
4. More likely to enroll
5. Much more likely to enroll

**[Q4] FOLLOW UP: TRAVEL TO AND FROM HOSPITAL**

QUESTION: If the research required you to **travel to and from a large hospital**, would this make you more or less likely to participate in this research study? You would be responsible for arranging your travel to and from the hospital.

Response options:

1. Much less likely to enroll
2. Less likely to enroll
3. Neutral
4. More likely to enroll
5. Much more likely to enroll

**[Q5] FOLLOW UP: RECEIVE CONTRAST AGENT**

QUESTION: If the research required **receiving an injection of a contrast material into your blood stream**, would this make you more or less likely to participate in the research study? The contrast material does not color organs, and does not contain radiation. The contrast liquid inserted into the blood stream enables the technology to create clearer pictures of your brain.

Response options:

1. Much less likely to enroll
2. Less likely to enroll
3. Neutral
4. More likely to enroll
5. Much more likely to enroll

**[Q6] FOLLOW UP: PRIVATE FIRM RESEARCHERS**

QUESTION: If the research was **conducted by researchers from a private, for-profit company** not associated with a university, would this make you more or less likely to participate in the research study?

Response options:

1. Much less likely to enroll
2. Less likely to enroll
3. Neutral
4. More likely to enroll
5. Much more likely to enroll

**[Q7] FOLLOW UP: FREE COPY OF BRAIN SCAN**

QUESTION: If the research team **sent you a copy of your brain scan images after your participation** would this make you more or less likely to participate in the research study?

Response options:

1. Much less likely to enroll
2. Less likely to enroll
3. Neutral
4. More likely to enroll
5. Much more likely to enroll

**[Q8] FOLLOW UP: FREE BRAIN HEALTH REPORT**

QUESTION: If participating in the research study **included sending you a brain health report based on the MRI images at no cost** would this make you more or less likely to participate in the research study?

Response options:

1. Much less likely to enroll
2. Less likely to enroll
3. Neutral
4. More likely to enroll
5. Much more likely to enroll

**[Q9] FOLLOW UP: NO MINORITIES ON RESEARCH TEAM**

QUESTION: If the research was **conducted by a research team that included racial or ethnic minority scientists** would you be more or less likely to participate in the research study?

Response options:

1. Much less likely to enroll
2. Less likely to enroll
3. Neutral
4. More likely to enroll
5. Much more likely to enroll

**[Q10] FOLLOW UP: NO COMMUNITY ENGAGEMENT**

QUESTION: If the research was **led by a research team that consulted with members of your community before conducting the study,** would you be more or less likely to participate in the research study?

Response options:

1. Much less likely to enroll
2. Less likely to enroll
3. Neutral
4. More likely to enroll
5. Much more likely to enroll

**[Q11a] BENEFITS OF MOBILE MRI – Closed Ended**

Note: The options presented in Column A are randomly ordered when presented to participants. And the “Benefits” and “Concerns” questions are randomly presented, i.e. some participants answer the Benefits questions before Concerns and vice versa.

In thinking about potentially participating in the research, please indicate how important, if at all, the following potential **BENEFITS** are to you:

|  | **Not important** | **Somewhat not important** | **Neither** | **Somewhat important** | **Very important** |
| --- | --- | --- | --- | --- | --- |
| Financial payment for time and travel | □ | □ | □ | □ | □ |
| Desire to help others and benefit future patients | □ | □ | □ | □ | □ |
| Contribute to scientific progress | □ | □ | □ | □ | □ |
| Interesting thing to do | □ | □ | □ | □ | □ |
| Access to medical treatment | □ | □ | □ | □ | □ |
| Learn more about your brain's health | □ | □ | □ | □ | □ |
| Get to see a cool picture of my brain scan, even if not part of a brain health report | □ | □ | □ | □ | □ |
| Learn if I have a medical condition that needs attention | □ | □ | □ | □ | □ |
| After the research is completed, get follow up information about the results of the study | □ | □ | □ | □ | □ |

**[Q11b] BENEFITS OF MOBILE MRI – Open Ended**

Note: The options presented in Column A are randomly ordered when presented to participants.

OPTIONAL -- If you wish to share any other benefits you might gain from the research, please type them here: ______________________________

**[Q12a] CONCERNS OF MOBILE MRI – Closed Ended**

Note: The options presented in Column A are randomly ordered when presented to participants.

In thinking about potentially participating in the research, please indicate how important, if at all, the following potential **CONCERNS** are to you:

|  | **Not important** | **Somewhat not important** | **Neither** | **Somewhat important** | **Very important** |
| --- | --- | --- | --- | --- | --- |
| Research team will not understand or respect my cultural and community values | □ | □ | □ | □ | □ |
| Researchers cannot be trusted / not sure of their motivations | □ | □ | □ | □ | □ |
| Brain scan might be used by insurance companies to raise my insurance rates | □ | □ | □ | □ | □ |
| Brain scan will not be safe | □ | □ | □ | □ | □ |
| Researchers will not respect my rights or privacy | □ | □ | □ | □ | □ |
| Researchers may use the brain scan for mind control | □ | □ | □ | □ | □ |
| Scared that I will find something wrong with my brain | □ | □ | □ | □ | □ |
| Not enough payment for time and travel | □ | □ | □ | □ | □ |
| Brain Scan would be too uncomfortable / I am claustrophobic | □ | □ | □ | □ | □ |
| Too busy and not enough time to participate in this research | □ | □ | □ | □ | □ |
| Metal or electronic device in my body | □ | □ | □ | □ | □ |

**[Q12b] BENEFITS OF MOBILE MRI – Open Ended**

Note: The options presented in Column A are randomly ordered when presented to participants.

OPTIONAL -- If you wish to share any other concerns you might have about this research, please type them here: ______________________________

**[Q13] FAMILIARITY WITH MRI**

How familiar are you with MRI? Please check all that apply.

Response options:

- I have had an MRI scan before.
- I know someone who has had an MRI scan before.
- I have heard of MRI scans before.
- I have seen an MRI scan before, for instance on a TV show, in a movie, or on a YouTube video.
- Other _______

**TRANSITION TEXT**

We would now like to ask you a question about **medical research in general.**

**[Q14] TRUST IN RESEARCH**

Note: These are verbatim questions from an instrument that has been used in the field. The full instrument is 12 questions long. I’ve just selected 3 here.

Please rate how strongly you agree or disagree with the following statements **about medical research generally**:

|  | **Strongly disagree** | **Disagree** | **Neutral** | **Agree** | **Strongly agree** |
| --- | --- | --- | --- | --- | --- |
| To get people to take part in a study, medical researchers usually do not explain all of the dangers about participation | □ | □ | □ | □ | □ |
| Usually, researchers who make mistakes try to cover them up | □ | □ | □ | □ | □ |
| Medical researchers act differently toward minority subjects than toward white  subjects | □ | □ | □ | □ | □ |

**ATTENTION FILTER**

There will be NO Attention Filter in the Verasight survey. See email discussion about this.

**[Q15] KNOWLEDGE CHECK 1 OF 3**

**Which study were you asked to consider joining?**

Response options:

1. A knee study using MRI
2. A Parkinson's disease study
3. A psychology study related to heart health
4. A brain study using MRI

**[Q16] KNOWLEDGE CHECK 2 OF 3**

How much would you be paid for the research study you were asked about participating in?

Response options:

1. $50
2. $100
3. $125
4. $150
5. $175
6. $200

**[Q17] KNOWLEDGE CHECK 3 OF 3**

Earlier you were presented with a picture of what the portable MRI machine could look like. In just a few words, what, if anything, do you remember about the picture you saw?

If you cannot remember anything, please type click “Don’t remember”.

Response options:

1. What I remember about the picture is: _____ [text box to fill in]
2. I don’t remember anything about the picture

**DEMOGRAPHICS**

Now we will ask you questions about your background.

**[Q18] GEOGRAPHY**

Which of the following best describes the place where you now live?

Response options:

1. Large city
2. Suburb near a large city
3. Small city or town
4. Rural area
5. Tribal lands

**[Q19] DISTANCE TO HOSPITAL**

If you had to travel to a major hospital, how long would it take you to travel there?

Response options:

1. Less than 15 minutes
2. 16 to 30 minutes
3. 31 to 60 minutes (1 hour)
4. 61 to 90 minutes
5. 91 to 120 minutes (2 hours)
6. More than 120 minutes (more than 2 hours)
7. Don’t know

**[Q20] HISPANIC / LATINO**

Do you consider yourself Hispanic or Latino?

Response options:

1. Yes
2. No

**[Q21] RACE**

Which of the following would you use to describe your race?

You can select as many as apply.

Response options:

1. Asian or Asian-American
2. Black or African-American
3. Hispanic / Latino
4. Native American / American Indian / Alaska Native
5. Pacific Islander / Native Hawaiian
6. White
7. Some other race ________

**[Q22] INCOME-1**

Last year, that is in 2021, what was your total household income from all sources, before taxes?

Response options:

1. Less than $50,000
2. $50,000 or more

**[Q23] INCOME-2**

Note: Only show this question if answer to INCOME-1 question is “Less than $50,000”

And was your household income last year…

Response options:

1. Less than $15,000
2. $15,000 to under $20,000
3. $20,000 to under $25,000
4. $25,000 to under $30,000
5. $30,000 to under $35,000
6. $35,000 to under $40,000
7. $40,000 to under $45,000
8. $45,000 to under $50,000

**[Q24] INCOME-3**

Note: Only show this question if answer to INCOME-1 question is “$50,000 or more”

And was your household income last year…

Response options:

1. $50,000 to under $75,000
2. $75,000 to under $100,000
3. $100,000 to under $150,000
4. $150,000 to under $200,000
5. More than $200,000

**[Q25] HOUSEHOLD SIZE**

Including yourself, how many people live in your household?

[OPEN NUMERIC ENTRY]

**[Q26] AGE**

Please enter your age in years:

[Drop down menu with ages]

**[Q27] GENDER**

Which best describes your gender?

Response options:

1. Male
2. Female
3. Other

**[Q28] EDUCATION**

What is the highest level of school that you have completed?

Response options:

1. Some high school or less
2. High school graduate or GED
3. Some college, no degree
4. 2-year or associate degree
5. 4-year or bachelor degree
6. Post-graduate degree

**[Q29] ZIP**

What is your ZIP code?

[Open numeric entry]

**[Q30] VOTE 2020**

Many Americans did not vote in the 2020 election for a variety of reasons, such as having to work or to take care of family members. Did you vote for president in the 2020 election?

Response options:

1. No, I did not vote
2. Yes, I voted for Joe Biden
3. Yes, I voted for Donald Trump
4. Yes, I voted for another candidate
5. Yes, I voted but prefer not to say which candidate

**[Q31] HEALTH INSURANCE COVERAGE**

Note: This wording is now consistent with the wording used in the U.S. Census Current Population Survey (CPS) health insurance module. The CPS module asks additional questions to parse out the details of the type of coverage, but that is not germane to our work here, so I’ve limited it to just these two questions.

**Are you CURRENTLY covered by any type of health plan?**

Response options:

1. Yes
2. No
3. Don’t Know

**Are you covered by Medicare?**

[only displays if they answer “Yes” to the question on health plan coverage.

Response options:

1. Yes
2. No
3. Don’t Know

**[Q32] RELATIONSHIP TO OLDER / VULNERABLE ADULT**

Which of the following best describes your relationship to an older or vulnerable adult who requires help making important decisions:

Response options:

1. I KNOW an older adult who needs help with decision-making
2. I am RELATED TO an older adult who needs help with decision-making
3. I LIVE WITH an older adult who needs help with decision-making
4. I am INVOLVED IN TAKING CARE OF an older adult who needs help with decision-making
5. Other (please explain) _________________

**[Q33] NEUROSCIENCE KNOWLEDGE**

How knowledgeable would you say you are about brain science in general?

Response options:

1 – Not at all knowledgeable

2 –

3 –

4 – Somewhat knowledgeable

5 –

6 –

7 – Very knowledgeable

**[Q34] END OF SURVEY DEBRIEF**

Thank you for participating in this study!

If you have any questions about this research project, please contact: [redacted]

If you have any further comments or questions about **research using portable brain scanning technologies**, please enter them in the box below:

**TO FINISH:** Click on the button below to submit your response and complete the survey.
